# Fast-acting antidepressant-like effects of ketamine in aged male rats

**DOI:** 10.1007/s43440-024-00636-y

**Published:** 2024-08-19

**Authors:** Elena Hernández-Hernández, Sandra Ledesma-Corvi, Jordi Jornet-Plaza, M. Julia García-Fuster

**Affiliations:** 1 IUNICS, University of the Balearic Islands, Cra. de Valldemossa, Km 7.5, Palma, E-07122 Spain; 2 Health Research Institute of the Balearic Islands (IdISBa), Palma, Spain; 3 Department of Medicine, University of the Balearic Islands, Palma, Spain; 4 Present address: Department of Pharmacology, University of the Basque Country (EHU/UPV), Leioa, Spain

**Keywords:** Ketamine, Fast-acting antidepressant, Aging, BDNF

## Abstract

**Background:**

The aging process causes anatomical and physiological changes that predispose to the development of late-life depression while reduces the efficacy of classical antidepressants. Novel fast-acting antidepressants such as ketamine might be good candidates to be explored in the context of aging, especially given the lack of previous research on its efficacy for this age period. Thus, the aim of the present study was to characterize ketamine’s effects in older rats.

**Methods:**

The fast-acting (30 min) and repeated (7 days) antidepressant-like effects of ketamine (5 mg/kg, *ip*) were evaluated in 14-month-old single-housed rats through the forced-swim and novelty-suppressed feeding tests. In parallel, the modulation of neurotrophic-related proteins (i.e., mBDNF, mTOR, GSK3) was assessed in brain regions affected by the aging process, prefrontal cortex and hippocampus, as well as possible changes in hippocampal cell proliferation.

**Results:**

Acute ketamine induced a fast-acting antidepressant-like response in male aged rats, as observed by a reduced immobility in the forced-swim test, in parallel with a region-specific increase in mBDNF protein content in prefrontal cortex. However, repeated ketamine failed to induce antidepressant-like efficacy, but decreased mBDNF protein content in prefrontal cortex. The rate of hippocampal cell proliferation and/or other markers evaluated was not modulated by either paradigm of ketamine.

**Conclusions:**

These results complement prior data supporting a fast-acting antidepressant-like effect of ketamine in rats, to further extend its efficacy to older ages. Future studies are needed to further clarify the lack of response after the repeated treatment as well as its potential adverse effects in aging.

**Supplementary Information:**

The online version contains supplementary material available at 10.1007/s43440-024-00636-y.

## Introduction

Major depressive disorder is one of the most common and debilitating mental illnesses and a major public health problem worldwide. After anxiety disorders, depression is the most common mental health disorder among older adults, affecting 5-7% of the elder population [1], with suicide rates also increasing between the ages of 60 and 90 [2]. Changes during aging predispose to the development of the so-called late-life depression [3], for which pharmacotherapy, psychotherapy, and electroconvulsive therapy are the treatments of choice [4]. However, classical antidepressants are known to change in efficacy with age [4–6], with reduced response rates ranging from 54% at age 54 to 42% at age 73 [6]. Despite these age-related differences, and in the context of our increasingly aging society, research focused on characterizing classical or novel therapeutic options for this age-group is really scarce (see some prior studies attempting to amend this [7–9]).

In this regard, the discovery of the rapid antidepressant effects of ketamine, an N-methyl-D-aspartate (NMDA) receptor antagonist, is considered one of the major breakthroughs in the treatment of depression in the past decades, opening the door to a new class of fast-acting antidepressant options [10]. While a single administration of ketamine has demonstrated rapid and potent reductions in depressive symptomatology both in humans [11–14] and in animal models of depression [15–20], repeated doses seemed to sustain the observed short-term responses [21–23]. Indeed, esketamine, the S-enantiomer of ketamine, was first approved by the FDA in US in 2019 and then by EMA in Europe for the treatment of patients with resistant depression [24–25]. Although efficacy in adult patients seemed quite satisfactory, the potential effects of ketamine to treat late-life depression has been poorly investigated. In particular, and as reviewed by [26–27], only two randomized controlled trials have been conducted in an aging population (60 years and older) [28–29], with mixed results. While one trial found a positive effect of ketamine administration both on response and remission rates [28], the other one found no significant differences [29]; although some beneficial effects were observed (i.e., a trend towards an improvement as measured by the Montgomery-Asberg Depression Rating Scale). Additionally, a recent analysis of an open-label clinical trial showed indications of a lower antidepressant response (37.1% vs. 57.8%) and remission rates (15.8% vs. 47.4%) in older vs. younger depressed patients [30]. These promising but clearly insufficient analyses highlight the need for increased research on the potential antidepressant-like effects of ketamine in the aging population, including its characterization at the preclinical level.

In this context and to the best of our knowledge, the evaluation of ketamine as an antidepressant in aged animals is limited to a single study conducted in mice evaluating the effects of a single administration [9], and another one evaluating its prophylactic effects against stress-induced behaviors [31]. However, neither of these studies [9, 31] induced efficacy in aged animals using a dosing regimen proven effective in adolescent and adult rodents (e.g., [19–20]). Against this background the present study will further characterize the potential antidepressant-like response of ketamine in aging single-housed rats (e.g., [32]), since this stress paradigm in rodents (i.e., social isolation due to size requirements for the number of animals per cage) reproduced several behavioral features mimicking late-life depression (see our own studies phenotyping rats from middle-age and onward [33–34, 8]). Moreover, the present study will evaluate biomarkers of the antidepressant-like response such as the activation of brain neurotrophic factor (BDNF) (e.g., [10, 35]) and associated signaling partners (e.g., mTOR and GSK-3; [36–38]) in several brain regions (e.g., prefrontal cortex and hippocampus) impacted by aging (e.g., [39]) and/or an increase in hippocampal neurogenesis [40–42]; see a more recent study by [43]). A preliminary report of a portion of this work was presented at the 34th European College of Neuropsychopharmacology (ECNP) Congress Hybrid [44].

## Materials and methods

### Animals

**Fig. 1:**
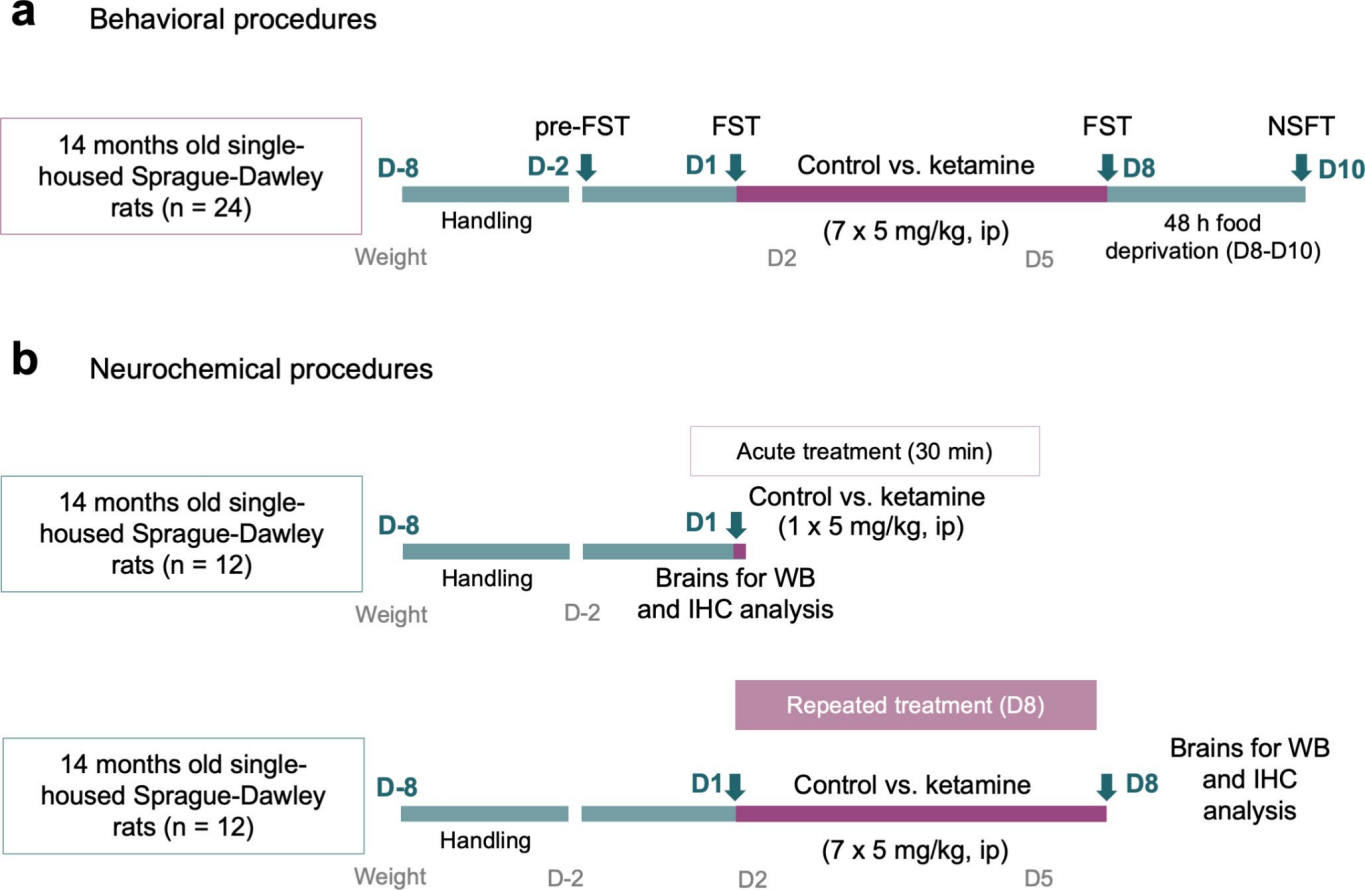
Experimental design. (**a**) Behavioral procedures in single-housed rats (14 months old) after an acute (1 dose of 5 mg/kg, i.p) or a repeated (7 doses of 5 mg/kg, *ip*, 1 dose per day) treatment with ketamine. Changes were evaluated 30-min (day, D1) or 24 h post-treatment (D8) in the forced-swim test (FST) and 3 days post-treatment (D10) in the novelty-suppressed feeding test (NSFT). (**b**) Neurochemical procedures aimed at collecting brains at the same time points (acute, D1, or repeated, D8) to evaluate molecular markers by western blot (WB) and immunohistochemistry (IHC) experiments

For the present study, a total of 48 male Sprague-Dawley rats were used when they reached 14-month-old. Rats were bred in the animal facility at the University of the Balearic Islands and were housed under standard vivarium conditions (22 ºC, 70% humidity, 12-h light/dark cycle, lights on at 8:00 AM) with access to a standard diet and unlimited tap water (except when otherwise specified, see Fig. 1a for food deprivation prior to novelty-suppressed feeding test). Following animal housing regulations regarding the number of animals allowed per cage in terms of size and weight, 14-month-old rats were single-housed in standard cages for several months before testing started, which is a great model of chronic stress. All procedures were performed during the light period (between 8:00 AM and 3:00 PM), complied with the ARRIVE guidelines [45], the EU Directive 2010/63/EU for animal experiments, and the Spanish Royal Decree 53/2013, and thus were approved by the Local Bioethical Committee (CEEA 58/04/16) and the Regional Government (2016/08/AEXP). All efforts were made to minimize the number of rats used and their suffering. Unfortunately, since no aged female rats were available at the time when this experiment was performed, only male rats were included in the present study, and therefore the conclusions are limited to only one sex.

### Behavioral procedures

All rats were handled several days prior to any behavioral or administration procedure. For this experiment, a total of 24 rats were used for the behavioral characterization of ketamine (Fig. 1a). Rats were randomly allocated into two groups and were treated daily for 7 consecutive days with ketamine (7 × 5 mg/kg, *ip*, *n* = 12; Anesketin: 100 mg/ml of ketamine from Dechra Pharmaceuticals, Northwich, United Kingdom) or saline (7 × 1 ml/kg of 0.9% NaCl, *ip*, *n* = 12). The dose of ketamine was chosen based on previous antidepressant results reported in the literature (e.g., [46–47]) and on our own results [19–20]. Antidepressant-like responses were ascertained by diverse tests previously validated in the field. We first measured behavioral despair under the stress of the forced-swim test (e.g., [48]), 30 min after the first treatment-dose (D1, acute effects) and 24 h after the last treatment dose (D8, repeated effects; see Fig. 1a) as earlier described by our group [33–34, 19]. Briefly, on pre-test day (D-2, see Fig. 1a), rats were placed for 15 min in single tanks (32 cm diameter x 41 cm high) filled with water (25 cm of depth, 25 ± 1 ºC). Then, on test days (D1 and D8) rats were exposed for 5 min to the same conditions, and their performances were videotaped. Videos were analyzed by an experimenter blind to the specific treatment groups with Behavioral Tracker software (CA, USA) to ascertain the time each rat spent (s) immobile (i.e., an indicative of despair) vs. active (i.e., escaping-like behaviors such as swimming or climbing). Moreover, the potential repeated antidepressant-like responses of ketamine were also evaluated in the novelty-suppressed feeding test 2 days after the last dose (D10). Following standard procedures [19, 49], rats were food-deprived for 48 h (D8-D10) since motivation for food is required for this particular test. During test day, each rat was placed for 5 min in a square open arena (60 cm x 60 cm, and 40 cm in high) under housing lighting conditions with three food pellets in the center [19]. Sessions were recorded and the parameters latency to center (s), time in center (s), latency to food (s), feeding time (s), and distance traveled (cm) were blindly scored using the ANY-maze software (version 7.37, Stoelting Co. Dublin, Ireland). Body weight was monitored trough the treatment process and showed no changes between experimental groups and/or across time (data not shown). Notably, brains from the behavioral procedures were not collected for neurochemical studies.

### Neurochemical procedures

For evaluating the neurochemical effects of ketamine, we utilized a separate group of 24 aged rats who were randomly assigned to the acute (1 dose; *n* = 12) or repeated (7 doses; *n* = 12) treatment groups (Fig. 1b). Similarly, to the previous experiment, each rat received the corresponding daily dose/es of saline (1 or 7 × 1 ml/kg of 0.9% NaCl, *ip*, *n* = 6 per group) or ketamine (1 or 7 × 5 mg/kg *ip*, *n* = 6 per group). Rats were then sacrificed without anesthesia by rapid decapitation with a large rodent guillotine following standard procedures in our group [8, 33] 30 min after the acute treatment or 24 h after last dose for the repeated treatment paradigm (D8). These time-points matched the particular times when forced-swim tests were performed so direct comparisons could be done between the potential antidepressant-like vs. neurochemical effects of ketamine in aged rats.

Once brains were rapidly extracted, the whole prefrontal cortex and the right hippocampus were freshly dissected, fast frozen in liquid nitrogen, and saved at -80 ºC until further processing to evaluate target proteins by Western blot analysis. The left hemisphere was frozen in -30 ºC isopentane (#143501,1611, Biolinea SL, Palma, Spain), and kept at -80 ºC until the entire hippocampal extent (-1.72 to -6.80 mm from Bregma) was cryostat-cut in 30 μm sections. Consecutive sections were slide mounted in 24 slides per animal with 8 tissue-sections per slide, divided in 3 series (8 slides per series), covering the most anterior part of the hippocampus, the middle part and the most posterior part of it. The rate of cell proliferation (Ki-67 + cells) was then evaluated by immunohistochemical analysis as previously performed [50–52] in a representative sample of the whole hippocampus following a stereological procedure that counts every 8-th section taken (1 slide from each series containing the anterior, middle and posterior part of hippocampus).

### Western blot analysis

Total homogenates of brain regions (prefrontal cortex or hippocampus) were prepared with minor modifications as previously described [53–54]. Each sample (40 µg of total protein) was loaded in 7.5–14% SDS-PAGE minigels (Bio-Rad Laboratories, Hercules, CA, USA) that were resolved by electrophoresis and then processed following standard immunoblotting procedures [33, 55]. Membranes (0.2 μm: #10600001 or 0.45 μm: #10600002, Merck SL, Barcelona, Spain) were incubated overnight at 4 ºC in blocking solution containing the specific primary antibodies: anti-BDNF (N-20) (dilution 1:10000; AB108319; Abcam, Cambridge, United Kingdom) for identifying the mature form of BDNF (mBDNF) [56]; anti-pS^2448^mTOR (dilution 1:1000; #2971) and anti-mTOR (dilution 1:1000; #2972) (Cell Signaling, MA, USA); anti-pS^21/9^-GSK3 (dilution 1:1000; # 9331; Cell Signaling) for detecting inhibitory phosphorylation and anti-GSK3 (dilution 1:1000; 4G-1E; 05-412; Millipore) for total protein. Membranes were then incubated with an anti-rabbit (#7074S) or anti-mouse (#7076S) horseradish peroxidase-conjugated secondary antibody (1:5000; Cell Signaling, MA, USA) and the immunoreactivity of target proteins was displayed on autoradiographic films (#28906837, Amersham ECL Hyperfilm) using an ECL detection system (#17652005, Amersham, Buckinghamshire, United Kingdom). Autoradiograms were quantified by densitometric scanning (GS-800 Imaging Calibrated Densitometer, Bio-Rad). All samples were loaded at least 2–3 times in different gels, and percent changes in each gel were calculated for each rat as compared to control-treated samples. For loading control, low quantities of total homogenates (15 µg) were run to detect β-actin (dilution 1:10000; AC-15; Sigma-Aldrich, MO, USA), since its content was not altered by the treatment followed.

### Immunohistochemical analysis

Cell proliferation was labeled in the hippocampus with Ki-67 antibody (1:20,000; from Prof. Huda Akil and Stanley J. Watson, University of Michigan, MI, USA) as previously described [50–52]. Experiments were performed on 3 cryostat-cut sections (30 µm), one from the front, middle or posterior parts of the hippocampus, and containing 8 tissue-sections per slide. Briefly, slide mounted sections were post-fixed in 4% paraformaldehyde and processed through several steps including a series of sequential incubation, with biotinylated anti-rabbit antibody (1:1000, BA-100, Vector Laboratories, CA, USA), Avidin/Biotin complex (PK-6100, Vectastain Elite ABC kit; Vector Laboratories, CA, USA) and the chromogen 3,3’-diaminobenzidine (H54000, Thermo Fischer, MA, USA) for signal detection. Finally, tissue was counterstained in cresyl-violet (405760100, Thermo Fischer, MA, USA), dehydrated in graded alcohols, submersed in xylene and cover-slipped with Permount^®^ (SP15-500, Fisher Chemical, NH, USA). The number of positive cells was counted by an experimenter blind to the treatment groups with a Leica DMR light microscope (63x objective lens) in a total of 3 slides per rat (8 sections per slide; total of 24 tissue sections per rat) and focusing through the depth of the tissue. The total number of cells was then multiplied by the sampling factor 8 to provide an overall estimate of the total number of Ki-67 + cells per rat in the left hippocampus.

### Data and statistical analysis

All data were analyzed and plotted with GraphPad Prism, Version 10 (GraphPad Software, Inc, San Diego, CA, USA). Results are expressed as mean values ± standard error of the mean (SEM); symbols represent individual values for each rat, following recommendations for displaying data and statistical results in pharmacology [57–58]. Potential changes induced by the acute or repeated treatments with ketamine for each behavioral feature and neurochemical marker analyzed were performed through two tailed Student’s *t*-tests. All sets of data reported followed a normal distribution according to Shapiro-Wilk normality test. The level of significance was set at *p* ≤ 0.05.

## Results

### Ketamine induced a rapid antidepressant-like response after an acute dose in aged rats: lack of efficacy following a repeated treatment

**Fig. 2:**
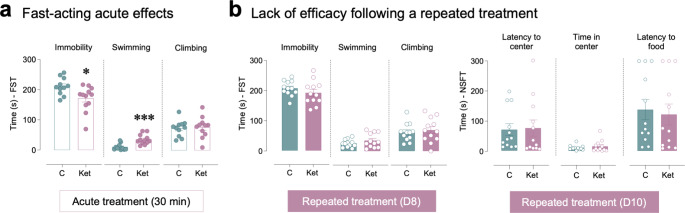
Exploring the antidepressant-like effects of ketamine in aged rats. (**a**) Fast-acting acute effects of ketamine (1 single dose of 5 mg/kg, *ip*, D1) as measured in the forced-swim test (FST) 30 min post-treatment. (**b**) Lack of efficacy following a repeated treatment with ketamine (5 mg/kg, *ip*, 7 days, D1-D7) as evaluated in the FST 24 h post-treatment (D8), and in the novelty-suppressed feeding test (NSFT) 3 days post-treatment (D10). Columns represent mean ± SEM of time spent in each behavior. Individual values are shown in symbols for each rat. ***p < 0.001, *p < 0.05 when comparing ketamine-treated rats (Ket) vs. control-treated rats (**C**) through two-tailed Student’s *t*-tests

When evaluating the acute antidepressant-like effects of ketamine under the stress of the forced-swim test, a two-tailed Student’s *t*-test revealed a significant reduction in immobility as observed 30 min post-ketamine administration (∼13% reduction: -40 ± 15 s, *t* = 2.62, *df* = 21, *p = 0.016 vs. control rats; Fig. 2a), which paralleled an increase in swimming behavior (∼8% increase: +25 ± 6 s, *t*_21_ = 4.29, ***p < 0.001 vs. control rats; Fig. 2a). Acute ketamine did not induce changes in climbing behavior (*t*_21_ = 0.87, *p* = 0.394; Fig. 2a).

Interestingly, following the repeated treatment with ketamine (7 days of a daily 5 mg/kg injection), no significant changes were observed in the forced-swim test (immobility: *t*_22_ = 1.14, *p* = 0.266; swimming: *t*_22_ = 1.20, *p* = 0.244; climbing: *t*_22_ = 0.50, *p* = 0.620) and/or the novelty-suppressed feeding test (latency to center: *t*_22_ = 0.17, *p* = 0.870; time in center: *t*_22_ = 0.77, *p* = 0. 452; latency to food: *t*_22_ = 0.33, *p* = 0.743), denotating the loss of antidepressant-like potential (Fig. 2b). Moreover, it is worth mentioning that none of the rats evaluated spend any feeding time (despite prior food deprivation) and no changes were observed in distance travelled among treatment groups (data not shown).

### Region-specific neurochemical effects after acute or repeated ketamine administration in aged rats

**Fig. 3:**
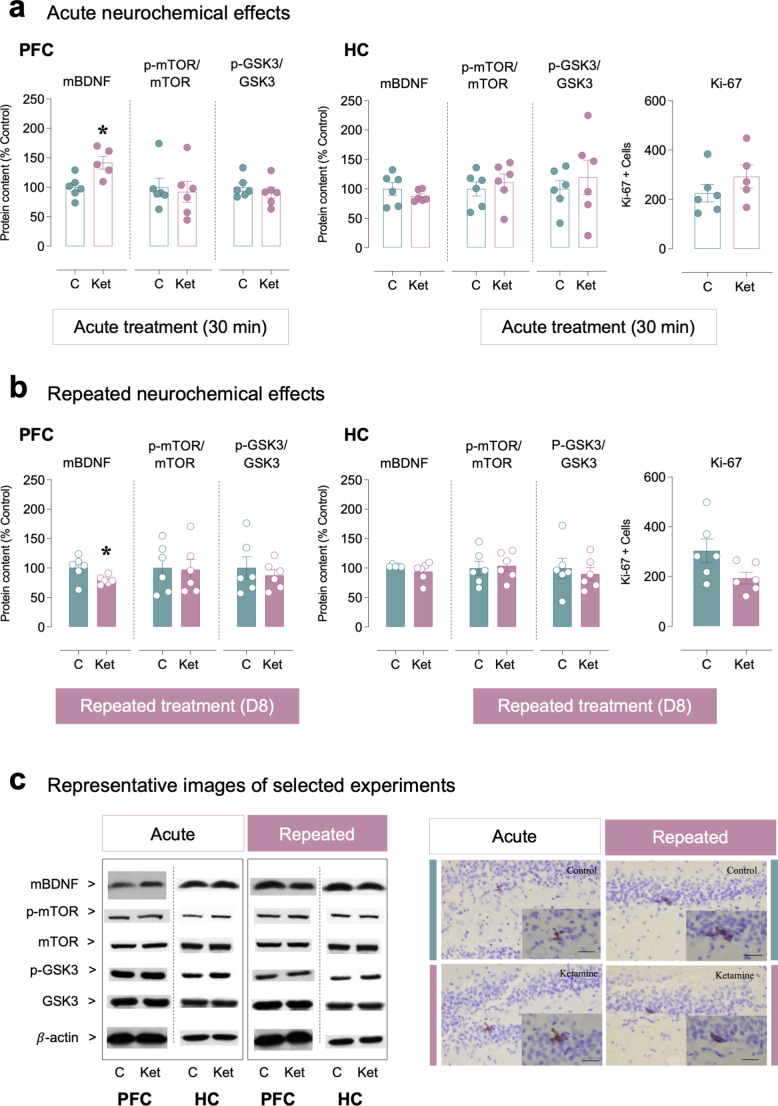
Exploring the neurochemical effects of ketamine in aged rats. (**a**) Fast-acting acute (30 min post-treatment) or (**b**) repeated (24 h post-treatment, D8) effects of ketamine in prefrontal cortex (PFC) and hippocampus (HC) of the selected protein markers evaluated by western blot (mBDNF, p-mTOR/mTOR and pGSK3/GSK3) or immunohistochemistry (Ki-67 + cells). Columns represent mean ± SEM of protein content (% Control) or Ki-67 + cells per group. Individual values are shown in symbols for each rat. *p < 0.05 when comparing ketamine-treated rats (Ket) vs. control-treated rats (**c**) through two-tailed Student’s *t*-tests. (**c**) Representative images of selected western blot or immunohistochemistry experiments. Left panels: immunoblots depicting the labeling of each protein and loading control β-actin. For unprocessed full western blot images check Supplementary Figures S1-S6. Right panels: representative images of Ki-67 + cells (brown labeling in the blue granular layer) taken with a light microscope using a 40x objective lens. A magnified window is shown at 63x. Scale bar: 30 μm. For other representative images check Supplementary Figures S7-S10

Acute ketamine induced a region-specific modulation of mBDNF protein content (Fig. 3a); while it increased mBDNF in prefrontal cortex (+ 42 ± 13%, *t*_9_ = 3.23, *p = 0.011 vs. control rats), no changes were observed in hippocampus (*t*_10_ = 1.07, *p* = 0.310). When analyzing the regulation of its downstream signaling partners (i.e., active ratio of p-mTOR/mTOR and inhibitory ratio of p-GSK3/GSK3), no changes were observed in prefrontal cortex or hippocampus (Fig. 3a). Moreover, acute ketamine did not change the rate of hippocampal cell proliferation (*t*_9_ = 1.17, *p* = 0.273).

The effects of a repeated paradigm of ketamine on the neurochemical markers under study are shown in Fig. 3b. The data showed that repeated ketamine in aged rats decreased the protein content of mBDNF in prefrontal cortex (-21 ± 9%, *t*_10_ = 2.35, *p = 0.041 vs. control rats), but not in hippocampus (*t*_9_ = 1.26, *p* = 0.239). Similar to what was observed following an acute injection, no changes were observed following a repeated paradigm in p-mTOR/mTOR or p-GSK3/GSK3 in both regions analyzed (Fig. 3b). Finally, repeated ketamine did not change the rate of hippocampal cell proliferation (*t*_10_ = 2.06, *p* = 0.067). Representative images of selected western blot and immunohistochemistry experiments are shown in Fig. 3c.

## Discussion

The present study investigated the acute and repeated antidepressant-like potential of ketamine in aged male rats following the same administration paradigms previously proven effective in adolescent and adult rats [19–20]. The main results showed a rapid fast-acting antidepressant-like effect of ketamine (observed 30 min post-treatment), as reported by a decrease in immobility and an increase in swimming behaviors in the forced-swim test, paired with an increase in mBDNF protein content in prefrontal cortex. However, these acute effects were no longer observed after a repeated treatment paradigm, as measured by a lack of antidepressant-like response in two independent behavioral tests, combined with a decrease in mBDNF in prefrontal cortex. These results proved an acute fast-acting antidepressant-like response for ketamine in aged male rats (observed both behaviorally and at the neurochemical level), while suggested that its repeated administration might lead to molecular adaptive changes preventing its efficacy.

Ketamine induced a rapid antidepressant-like response, observed 30 min after a single administration in aged rats, consistent with previous studies [15–16]. Interestingly our prior studies which utilized the same acute dose of ketamine (5 mg/kg) showed differences in antidepressant-like efficacy depending on the age of animals, the biological sex and prior stress exposure [19–20]. Particularly, acute ketamine induced an antidepressant-like response in adolescent rats in the forced-swim test, an effect that was observed for both sexes, but that depended on prior stress exposure (see further details in [19]). However, acute ketamine in adult rats was inefficacious for both sexes and only showed efficacy when male rats were priory pretreated with letrozol (an aromatase inhibitor that blocks the biosynthesis of estrogens; [20]). Overall, these results suggest that there may be some similarities in the antidepressant-like response between adolescent and aged rats, with a similar dose needed in aged [8] and adolescent rats, but with the need for a higher dose to induce efficacy in adult rats [59]. Remarkably, it is worth mentioning that the parallelisms observed for adolescent and aged rats were under different sources of stress (i.e., early maternal separation for the adolescent study [19] vs. social isolation due to size and cage requirements in the present study with aged rats), suggesting that ketamine proved good efficacy for stress-related conditions. In particular, the present experimental paradigm of physiologically aged rats, individually housed for several months, and with an expected phenotype mimicking depressive-like manifestations (see characterization at [33]) proves to be a great model in which to validate the fast-acting acute effects of ketamine administration.

Contrarily to prior reports demonstrating that under stressful situations repeated ketamine induced antidepressant-like effects in adolescent [19] and/or adult rats [20, 23, 60–64], the present results showed a lack of response in aged male rats. Disparities in the type of stressor and/or differences in ketamine pharmacokinetics due to age might be behind these discrepancies, as studies with anesthetic doses of ketamine showed a significant increase in half-life, drug availability, and duration of anesthesia in aged Sprague-Dawley rats compared to young rats [62]. Although test repetition might have been behind the lack of effects observed following the repeated paradigm, this seems unlikely, since we followed a standard procedure previously used in other studies from our group that had proven effects across time in the forced-swim test (e.g., [19–20, 52]). Moreover, similar to the low dose we tested (5 mg/kg), the same dose also rendered inefficacious in another study [61], suggesting dose-dependent effects as mentioned in some of the other reports, and the potential need for a much higher dose and/or an increasing-dosage regimen to observe a beneficial response after a repeated treatment, and overcome potential adaptive mechanisms. For example, pharmacodynamic responses caused by the daily repeated administration might be playing a role in this lack of efficacy (i.e., tachyphylaxis). In this context, we aimed at exploring the differences in the molecular responses elicited after an acute or repeated dosing paradigm in an attempt to further understand the behavioral results. Particularly, we explored markers of antidepressant-like responses (i.e., mBDNF and associated partners, as well as the first stage of hippocampal neurogenesis) in key brain areas mediating affective-like responses and impacted by the aging process (i.e., prefrontal cortex and/or hippocampus) (see [63–64] and references therein).

Concurrent with the observed fast-acting antidepressant-like response, a single administration of ketamine increased mBDNF expression in the prefrontal cortex of aged male rats. This effect was not observed in hippocampus, suggesting a region-specific role for prefrontal cortex in the molecular actions behind ketamine’s response, and in line with prior results (reviewed by [35] and recently by [10]). BDNF-mediated activation of tropomyosin receptor kinase B (TrkB) induces the activation of several signaling pathways, including the inhibitory phosphorylation of the glycogen synthase kinase-3 (GSK-3) [36–37], which ultimately activates the mechanistic target of rapamycin complex 1 (mTORC1) [38]. However, no changes were detected in mBDNF-associated downstream partners in either brain region under evaluation (i.e., p-mTOR/mTOR and p-GSK3/GSK3 ratios). Interestingly, and in line with the present results, increased BDNF expression in the prefrontal cortex has been shown to be necessary for the acute antidepressant-like effects of ketamine [10, 65]). However, during aging, changes in TrkB expression may be involved in the lack of downstream signaling [66]. Also, since ketamine activates mTOR signaling within 1 h in the prefrontal cortex [67], another possible explanation for these results could be that the chosen time point of study might be too early to observe changes in the TrkB downstream pathway.

Curiously, repeated ketamine treatment reduced mBDNF expression in the prefrontal cortex as evaluated 24 h after the last daily dose, without altering its content in hippocampus and/or its downstream partners and hippocampal cell proliferation. These effects could be related to the lack of antidepressant-like efficacy after repeated ketamine administration and might imply certain adaptive molecular changes to the acute increase in mBDNF and caused by the repeated administration of the drug. Moreover, since some studies in the literature described that the sustained antidepressant ketamine response seemed to require hippocampal progenitor differentiation through a TrkB-dependent mechanism [68], the observed lack of BDNF modulation in hippocampus after repeated ketamine treatment may predict its lack of effect on cell proliferation. Moreover, previous studies have shown that aging abolishes the neurogenic effect of classical (e.g., fluoxetine; [69]) and novel antidepressants (e.g., cannabidiol [8]), possibly due to changes in the neurogenic niche during aging [70].

A limitation of the present study is the fact that it was conducted exclusively in male rats, especially since depression is twice as common in women as in men [71] and given that ketamine has demonstrated sex-dependent antidepressant-like effects in rats of different ages [46, 19–20]. Unfortunately, the logistical burden of individually housing animals as they age for several months severely hampered the inclusion of sex as a biological variable in the present study. Therefore, future studies should ascertain sex differences when characterizing the potential antidepressant-like effects of ketamine in aging, as preclinical animal models must be truly representative of the aging population. Of special relevance would be to ascertain how potential sex differences regarding the functioning of the glutamate system (e.g., reviewed by [72]), and especially the NMDA receptor [73] might be affecting the antidepressant-like response of ketamine in aged male vs. female rats.

In conclusion, our study contributes by increasing the existing body of knowledge on the role of ketamine as an antidepressant through its effects in aged rats. Overall, acute ketamine administration showed a fast-acting antidepressant-like response in aged rats (behavioral and biomarker responses). Future studies are needed to clarify the lack of response after the repeated treatment as well as its potential adverse effects. Moreover, other aspects to further study when trying to find a safe and effective treatment for age-related depression would include characterizing the duration of the acute antidepressant-like response, evaluating other doses and/or administration regimens, as well as including sex as a biological variable. In any case, ketamine seems like a great novel fast-acting option to be further explored for our aged population in which classical antidepressants showed reduced efficacy.

## Electronic supplementary material

Below is the link to the electronic supplementary material.

Supplementary Material 1

## Data Availability

Data will be made available upon request.

## References

[1] World Health Organization (WHO). (2024, may). Depressive disorder (depression). Available online: https://www.who.int/news-room/fact-sheets/detail/depression

[2] Shah A, Bhat R, Zarate-Escudero S, Deleo D, Erlangsen A. Suicide rates in five-year age-bands after the age of 60 years: the international landscape. Aging Ment Health. 2016;20:131–8. 26094783 10.1080/13607863.2015.1055552

[3] McKinney BC, Sibille E. (2013). The age-by-disease interaction hypothesis of late-life depression. Am J Geriatr Psychiatry. 2013;21:418–432. 10.1016/j.jagp.2013.01.053 PMC3549303 23570886

[4] Alexopoulos GS. Mechanisms and treatment of late-life depression. Transl Psychiatry. 2019;9:188. 31383842 10.1038/s41398-019-0514-6 PMC6683149

[5] Felice D, O’Leary OF, Cryan JF, Dinan TG, Gardier AM, Sánchez C, et al. When ageing meets the blues: are current antidepressants effective in depressed aged patients? Neurosci Biobehav Rev. 2015;55:478–97. 26054791 10.1016/j.neubiorev.2015.06.005

[6] Tedeschini E, Levkovitz Y, Iovieno N, Ameral VE, Nelson JC, Papakostas GI. Efficacy of antidepressants for late-life depression: a meta-analysis and meta-regression of placebo-controlled randomized trials. J Clin Psychiatry. 2011;72:1660–8. 22244025 10.4088/JCP.10r06531

[7] Fernández-Guasti A, Olivares-Nazario M, Reyes R, Martínez-Mota L. Sex and age differences in the antidepressant-like effect of fluoxetine in the forced swim test. Pharmacoly Biochem Behav. 2017;152:81–9. 10.1016/j.pbb.2016.01.011 26807812

[8] Hernández-Hernández E, García-Fuster MJ. Dose-dependent antidepressant-like effects of cannabidiol in aged rats. Front Pharmacol. 2022;13:891842. 35847003 10.3389/fphar.2022.891842 PMC9283859

[9] Mastrodonato A, Pavlova I, Kee N, McGowan JC, Mann JJ, Denny CA. Acute (R,S)-ketamine administration induces sex-specific behavioral effects in adolescent but not aged mice. Front Neurosci. 2022;16:852010. 35527817 10.3389/fnins.2022.852010 PMC9069103

[10] Deyama S, Kaneda K. Role of neurotrophic and growth factors in the rapid and sustained antidepressant actions of ketamine. Neuropharmacology. 2023;224:109335. 36403852 10.1016/j.neuropharm.2022.109335

[11] Berman RM, Cappiello A, Anand A, Oren DA, Heninger GR, Charney DS, et al. Antidepressant effects of ketamine in depressed patients. Biol Psychiatry. 2000;47:351–4. 10686270 10.1016/S0006-3223(99)00230-9

[12] Hu YD, Xiang YT, Fang JX, Zu S, Sha S, Shi H et al. (2016). Single i.v. ketamine augmentation of newly initiated escitalopram for major depression: Results from a randomized, placebo-controlled 4-week study. Psychol Med. 2016;46:623–635. 10.1017/S0033291715002159 26478208

[13] Ionescu DF, Luckenbaugh DA, Niciu MJ, Richards EM, Zarate CA. A single infusion of ketamine improves depression scores in patients with anxious bipolar depression. Bipolar Disord. 2015;17:438–43. 25400146 10.1111/bdi.12277 PMC4431955

[14] Wilkinson ST, Ballard ED, Bloch MH, Mathew SJ, Murrough JW, Feder A et al. (2018). The effect of a single dose of intravenous ketamine on suicidal ideation: A systematic review and individual participant data meta-analysis. Am J Psychiatry. 2018;175:150–158. 10.1176/appi.ajp.2017.17040472 PMC5794524 28969441

[15] Autry AE, Adachi M, Nosyreva E, Na ES, Los MF, Cheng PF, et al. NMDA receptor blockade at rest triggers rapid behavioural antidepressant responses. Nature. 2011;475:91–6. 21677641 10.1038/nature10130 PMC3172695

[16] Franceschelli A, Sens J, Herchick S, Thelen C, Pitychoutis PM. Sex differences in the rapid and the sustained antidepressant-like effects of ketamine in stress-naïve and depressed mice exposed to chronic mild stress. Neuroscience. 2015;290:49–60. 25595985 10.1016/j.neuroscience.2015.01.008

[17] Fitzgerald PJ, Yen JY, Watson BO. Stress-sensitive antidepressant-like effects of ketamine in the mouse forced swim test. PLoS ONE. 2019;14:e0215554. 30986274 10.1371/journal.pone.0215554 PMC6464213

[18] Wilson C, Li S, Hannan AJ, Renoir T. Antidepressant-like effects of ketamine in a mouse model of serotonergic dysfunction. Neuropharmacology. 2020;168:107998. 32061666 10.1016/j.neuropharm.2020.107998

[19] Ledesma-Corvi S, Hernández-Hernández E, García-Fuster MJ. Exploring pharmacological options for adolescent depression: a preclinical evaluation with a sex perspective. Transl Psychiatry. 2022;12:220. 35650182 10.1038/s41398-022-01994-y PMC9160287

[20] Ledesma-Corvi S, Jornet-Plaza J, García-Fuster MJ. Aromatase inhibition and ketamine in rats: sex-differences in antidepressant-like efficacy. Biol Sex Diff. 2023;14:1–13. 10.1186/s13293-023-00560-5 PMC10599051 37876000

[21] Ghasemi M, Kazemi MH, Yoosefi A, Ghasemi A, Paragomi P, Amini H et al. (2014). Rapid antidepressant effects of repeated doses of ketamine compared with electroconvulsive therapy in hospitalized patients with major depressive disorder. Psychiatry Res. 2014;215:355–361. 10.1016/j.psychres.2013.12.008 24374115

[22] Murrough JW, Perez AM, Pillemer S, Stern J, Parides MK, Aan Het Rot M, et al. Rapid and longer-term antidepressant effects of repeated ketamine infusions in treatment-resistant major depression. Biol Psychiatry. 2013;74:250–6. 22840761 10.1016/j.biopsych.2012.06.022 PMC3725185

[23] Thelen C, Sens J, Mauch J, Pandit R, Pitychoutis PM. Repeated ketamine treatment induces sex-specific behavioral and neurochemical effects in mice. Behav Brain Res. 2016;312:305–12. 27343934 10.1016/j.bbr.2016.06.041

[24] Kim J, Farchione T, Potter A, Chen Q, Temple R. Esketamine for treatment-resistant depression — first FDA-approved antidepressant in a new class. N Engl J Med. 2019;381:1–4. 31116916 10.1056/NEJMp1903305

[25] Mahase E. Esketamine is approved in Europe for treating resistant major depressive disorder. BMJ. 2019;367:I7069. 10.1136/bmj.l7069 31862692

[26] Di Vincenzo JD, Siegel A, Lipsitz O, Ho R, Teopiz KM, Ng J, et al. The effectiveness, safety and tolerability of ketamine for depression in adolescents and older adults: a systematic review. J Psychiatri Res. 2021;137:232–41. 10.1016/j.jpsychires.2021.02.058 33706168

[27] Gupta A, Dhar R, Patadia P, Funaro M, Bhattacharya G, Farheen SA et al. (2021). A systematic review of ketamine for the treatment of depression among older adults. Int Psychogeriatr. 2021;33:179–191. 10.1017/S1041610220000903 32600480

[28] George D, Gálvez V, Martin D, Kumar D, Leyden J, Hadzi-Pavlovic D, et al. Pilot randomized controlled trial of titrated subcutaneous ketamine in older patients with treatment-resistant depression. Am J Geriatr Psychiatry. 2017;25:1199–209. 28739263 10.1016/j.jagp.2017.06.007

[29] Ochs-Ross R, Daly EJ, Zhang Y, Lane R, Lim P, Morrison RL et al. (2020). Efficacy and safety of esketamine nasal spray plus an oral antidepressant in elderly patients with treatment-resistant depression—TRANSFORM-3. Am J Geriatr Psychiatry. 2020;28:121–141. 10.1016/j.jagp.2019.10.008 31734084

[30] Zheng W, Zhou YL, Wang CY, Lan XF, Ning YP. (2023). A comparative analysis of antidepressant and anti-suicidal effects of repeated ketamine infusions in elderly and younger adults with depression. J Affect Disorde. 2023;334:145–151. 10.1016/j.jad.2023.04.120 37160235

[31] Mastrodonato A, Pavlova I, Kee N, Pham VA, McGowan JC, Mann JJ, et al. Prophylactic (R,S)-ketamine is effective against stress-induced behaviors in adolescent but not aged mice. Int J Neuropsychopharmacol. 2022;25:512–23. 35229871 10.1093/ijnp/pyac020 PMC9211010

[32] Herrera-Pérez JJ, Martínez-Mota L, Fernández-Guasti A. Aging increases the susceptibility to develop anhedonia in male rats. Prog Neuropsychopharmacol Biol Psychiatry. 2008;32:1798–803. 18722496 10.1016/j.pnpbp.2008.07.020

[33] Hernández-Hernández E, Miralles A, Esteban S, García-Fuster MJ. Improved age-related deficits in cognitive performance and affective-like behavior following acute, but not repeated, 8-OH-DPAT treatments in rats: regulation of hippocampal FADD. Neurobiol Aging. 2018;71:115–26. 30138765 10.1016/j.neurobiolaging.2018.07.014

[34] Hernández-Hernández E, García-Fuster MJ. Evaluating the effects of 2-BFI and tracizoline, two potent I2-imidazoline receptor agonists, on cognitive performance and affect in middle-aged rats. Naunyn Schmiedebergs Arch Pharmacol. 2021;394:898–996. 10.1007/s00210-020-02042-6 33415506

[35] Björkholm C, Monteggia LM. BDNF - A key transducer of antidepressant effects. Neuropharmacology. 2016;102:72–9. 26519901 10.1016/j.neuropharm.2015.10.034 PMC4763983

[36] Beurel E, Song L, Jope RS. Inhibition of glycogen synthase kinase-3 is necessary for the rapid antidepressant effect of ketamine in mice. Mol Psychiatry. 2011;16:1068–70. 21502951 10.1038/mp.2011.47 PMC3200424

[37] Liu RJ, Fuchikami M, Dwyer JM, Lepack AE, Duman RS, Aghajanian GK. GSK-3 inhibition potentiates the synaptogenic and antidepressant-like effects of subthreshold doses of ketamine. Neuropsychopharmacology. 2013;38:2268–77. 23680942 10.1038/npp.2013.128 PMC3773678

[38] Zanos P, Gould TD. Mechanisms of ketamine action as an antidepressant. Mol Psychiatry. 2018;23:801–11. 29532791 10.1038/mp.2017.255 PMC5999402

[39] Rosenzweig ES, Barnes CA. (2003). Impact of aging on hippocampal function: Plasticity, network dynamics, and cognition. Prog Neurobiol. 2003;69:143–179. 10.1016/s0301-0082(02)00126-0 12758108

[40] Malberg JE, Eisch AJ, Nestler EJ, Duman RS. Chronic antidepressant treatment increases neurogenesis in adult rat hippocampus. J Neurosci. 2000;20:9104–10. 11124987 10.1523/JNEUROSCI.20-24-09104.2000 PMC6773038

[41] Malberg JE, Duman RS. Cell proliferation in adult hippocampus is decreased by inescapable stress: reversal by fluoxetine treatment. Neuropsychopharmacology. 2003;28:1562–71. 12838272 10.1038/sj.npp.1300234

[42] Keilhoff G, Bernstein HG, Becker A, Grecksch G, Wolf G. Increased neurogenesis in a rat ketamine model of schizophrenia. Biol Psychiatry. 2004;56(5):317–22. 15336513 10.1016/j.biopsych.2004.06.010

[43] Rawat R, Tunc-Ozcan E, Dunlop S, Tsai YH, Li F, Bertossi R, et al. Ketamine’s rapid and sustained antidepressant effects are driven by distinct mechanisms. Cell Mol Life Sci. 2024;81:105. 38413417 10.1007/s00018-024-05121-6 PMC10899278

[44] Hernández-Hernández E, Ledesma-Corvi S, García-Fuster MJ. Evaluation of the antidepressant-like potential of ketamine in aged rats. Eur Neuropsychopharmacol. 2021;53:S278–9. 10.1016/j.euroneuro.2021.10.361

[45] Percie du Sert N, Hurst V, Ahluwalia A, Alam S, Avey MT, Baker M et al. (2020). The ARRIVE guidelines 2.0: Updated guidelines for reporting animal research. Br J Pharmacol. 2020;177:3617–3624. 10.1111/bph.15193 PMC7393194 32662519

[46] Sarkar A, Kabbaj M. Sex differences in effects of ketamine on behavior, spine density, and synaptic proteins in socially isolated rats. Biol Psychiatry. 2016;80:448–56. 26957131 10.1016/j.biopsych.2015.12.025 PMC4940294

[47] Hibicke M, Landry AN, Kramer HM, Talman ZK, Nichols CD. Psychedelics, but not ketamine, produce persistent antidepressant-like effects in a rodent experimental system for the study of depression. ACS Chem Neurosci. 2020;11:864–71. 32133835 10.1021/acschemneuro.9b00493

[48] Cryan JF, Markou A, Lucki I. Assessing antidepressant activity in rodents: recent developments and future needs. Trends Pharmacol Sci. 2002;23:238–45. 12008002 10.1016/S0165-6147(02)02017-5

[49] Turner CA, Gula EL, Taylor LP, Watson SJ, Akil H. Antidepressant-like effects of intracerebroventricular FGF2 in rats. Brain Res. 2008;1224:63–8. 18586016 10.1016/j.brainres.2008.05.088 PMC2532793

[50] García-Fuster MJ, Perez JA, Clinton SM, Watson SJ, Akil H. Impact of cocaine on adult hippocampal neurogenesis in an animal model of differential propensity to drug abuse. Eur J Neurosci. 2010;31:79–89. 20104651 10.1111/j.1460-9568.2009.07045.x PMC4037740

[51] García-Fuster MJ, Flagel SB, Mahmood ST, Mayo LM, Thompson RC, Watson SJ, et al. Decreased proliferation of adult hippocampal stem cells during cocaine withdrawal: possible role of the cell fate regulator FADD. Neuropsychopharmacology. 2011;36:2303–17. 21796105 10.1038/npp.2011.119 PMC3176567

[52] García-Cabrerizo R, Ledesma-Corvi S, Bis-Humbert C, García-Fuster MJ. Sex differences in the antidepressant-like potential of repeated electroconvulsive seizures in adolescent and adult rats: regulation of the early stages of hippocampal neurogenesis. Eur Neuropsychopharmacol. 2020;41:132–45. 33160794 10.1016/j.euroneuro.2020.10.008

[53] García-Fuster MJ, Miralles A, García-Sevilla JA. Effects of opiate drugs on Fas-associated protein with death domain (FADD) and effector caspases in the rat brain: regulation by the ERK1/2 MAP kinase pathway. Neuropsychopharmacology. 2007;32:399–411. 16482086 10.1038/sj.npp.1301040

[54] García-Fuster MJ, García-Sevilla JA. Effects of anti-depressant treatments on FADD and p-FADD protein in rat brain cortex: enhanced anti-apoptotic p-FADD/FADD ratio after chronic desipramine and fluoxetine administration. Psychopharmacology. 2016;233:2955–71. 27259485 10.1007/s00213-016-4342-6

[55] Salort G, Hernández-Hernández E, García-Fuster MJ, García-Sevilla JA. Regulation of cannabinoid CB1 and CB2 receptors, neuroprotective mTOR and pro-apoptotic JNK1/2 kinases in postmortem prefrontal cortex of subjects with major depressive disorder. J Affect Disord. 2020;276:626–35. 32871695 10.1016/j.jad.2020.07.074

[56] Diniz CRAF, Casarotto PC, Resstel L, Joca SRL. Beyond good and evil: a putative continuum-sorting hypothesis for the functional role of proBDNF/BDNF-propeptide/mBDNF in antidepressant treatment. Neurosci Biobeh Rev. 2018;90:70–83. 10.1016/j.neubiorev.2018.04.001 29626490

[57] Curtis MJ, Alexander S, Cirino G, Docherty JR, George CH, Giembycz MA, et al. Experimental design and analysis and their reporting II: updated and simplified guidance for authors and peer reviewers. Br J Pharmacol. 2018;175:987–93. 29520785 10.1111/bph.14153 PMC5843711

[58] Michel MC, Murphy TJ, Motulsky HJ. (2020). New author guidelines for displaying data and reporting data analysis and statistical methods in experimental biology. J Pharmacol Exp Ther. 2020;372:136–147. 10.1124/jpet.119.264143 31884418

[59] Bis-Humbert C, García-Cabrerizo R, García-Fuster MJ. Decreased sensitivity in adolescent versus adult rats to the antidepressant-like effects of cannabidiol. Psychopharmacology. 2020;237:1621–31. 32086540 10.1007/s00213-020-05481-4

[60] Garcia LSB, Comim CM, Valvassori SS, Réus GZ, Stertz L, Kapczinski F, et al. Ketamine treatment reverses behavioral and physiological alterations induced by chronic mild stress in rats. Prog Neuropsychopharmacol Biol Psychiatry. 2009;33:450–5. 19439250 10.1016/j.pnpbp.2009.01.004

[61] Parise EM, Alcantara LF, Warren BL, Wright KN, Hadad R, Sial OK, et al. Repeated ketamine exposure induces an enduring resilient phenotype in adolescent and adult rats. Biol Psychiatry. 2013;74:750–9. 23790225 10.1016/j.biopsych.2013.04.027 PMC3785550

[62] Veilleux-Lemieux D, Castel A, Carrier D, Beaudry F, Vachon P. Pharmacokinetics of ketamine and xylazine in young and old Sprague-Dawley rats. J Am Assoc Lab Anim Sci. 2013;52:567–70. 24041212 PMC3784662

[63] Jaggar M, Fanibunda SE, Ghosh S, Duman RS, Vaidya VA. The neurotrophic hypothesis of depression revisited: new insights and therapeutic implications. Neurobiology of Depression: Road to Novel therapeutics (pp. 43–62). Elsevier.

[64] Deyama S, Duman RS. Neurotrophic mechanisms underlying the rapid and sustained antidepressant actions of ketamine. Pharmacol Biochem Behav. 2020;188:172837. 31830487 10.1016/j.pbb.2019.172837 PMC6997025

[65] Lepack AE, Fuchikami M, Dwyer JM, Banasr M, Duman RS. BDNF release is required for the behavioral actions of ketamine. Int J Neuropsychopharmacol. 2014;18:1–6. 10.1093/ijnp/pyu033 PMC4368871 25539510

[66] Dwivedi Y. Involvement of brain-derived neurotrophic factor in late-life depression. Am J Geriatr Psychiatry. 2013;21:433–49. 23570887 10.1016/j.jagp.2012.10.026 PMC3767381

[67] Li N, Lee B, Liu R-J, Banasr M, Dwyer JM, Iwata M, et al. mTOR-Dependent synapse formation underlies the rapid antidepressant effects of NMDA antagonists. Science. 2010;329:959–64. 20724638 10.1126/science.1190287 PMC3116441

[68] Ma Z, Zang T, Birnbaum SG, Wang Z, Johnson JE, Zhang CL, et al. TrkB dependent adult hippocampal progenitor differentiation mediates sustained ketamine antidepressant response. Nat Commun. 2017;8:1668. 29162814 10.1038/s41467-017-01709-8 PMC5698402

[69] Couillard-Despres S, Wuertinger C, Kandasamy M, Caioni M, Stadler K, Aigner R, et al. Ageing abolishes the effects of fluoxetine on neurogenesis. Mol Psychiatry. 2009;14:856–64. 19139747 10.1038/mp.2008.147

[70] Mosher KI, Schaffer DV. Influence of hippocampal niche signals on neural stem cell functions during aging. Cell Tissue Res. 2018;371:115–24. 29124394 10.1007/s00441-017-2709-6 PMC5750097

[71] Labaka A, Goñi-Balentziaga O, Lebeña A, Pérez-Tejada J. Biological Sex differences in depression: a systematic review. Biol Res Nurs. 2018;20:383–92. 29759000 10.1177/1099800418776082

[72] Giacometti LL, Barker JM. Sex differences in the glutamate system: implications for addiction. Neurosci Biobehav Rev. 2020;113:157–68. 32173404 10.1016/j.neubiorev.2020.03.010 PMC7225077

[73] Torrisi SA, Rizzo S, Laudani S, Ieraci A, Drago F, Leggio GM. Acute stress alters recognition memory and AMPA/NMDA receptor subunits in a sex-dependent manner. Neurobiol Stress. 2023;25:100545. 37293561 10.1016/j.ynstr.2023.100545 PMC10244889

