# Supplementary material for: Fast-acting antidepressant-like effects of ketamine in aged male rats
Source: Pharmacol Rep. 2024 Aug 19;76(5):991–1000. doi: 10.1007/s43440-024-00636-y (PMC11387441; doi:10.1007/s43440-024-00636-y)
Supplement: Supplementary file 1 — Supplementary Material 1 [file 43440_2024_636_MOESM1_ESM.pdf]

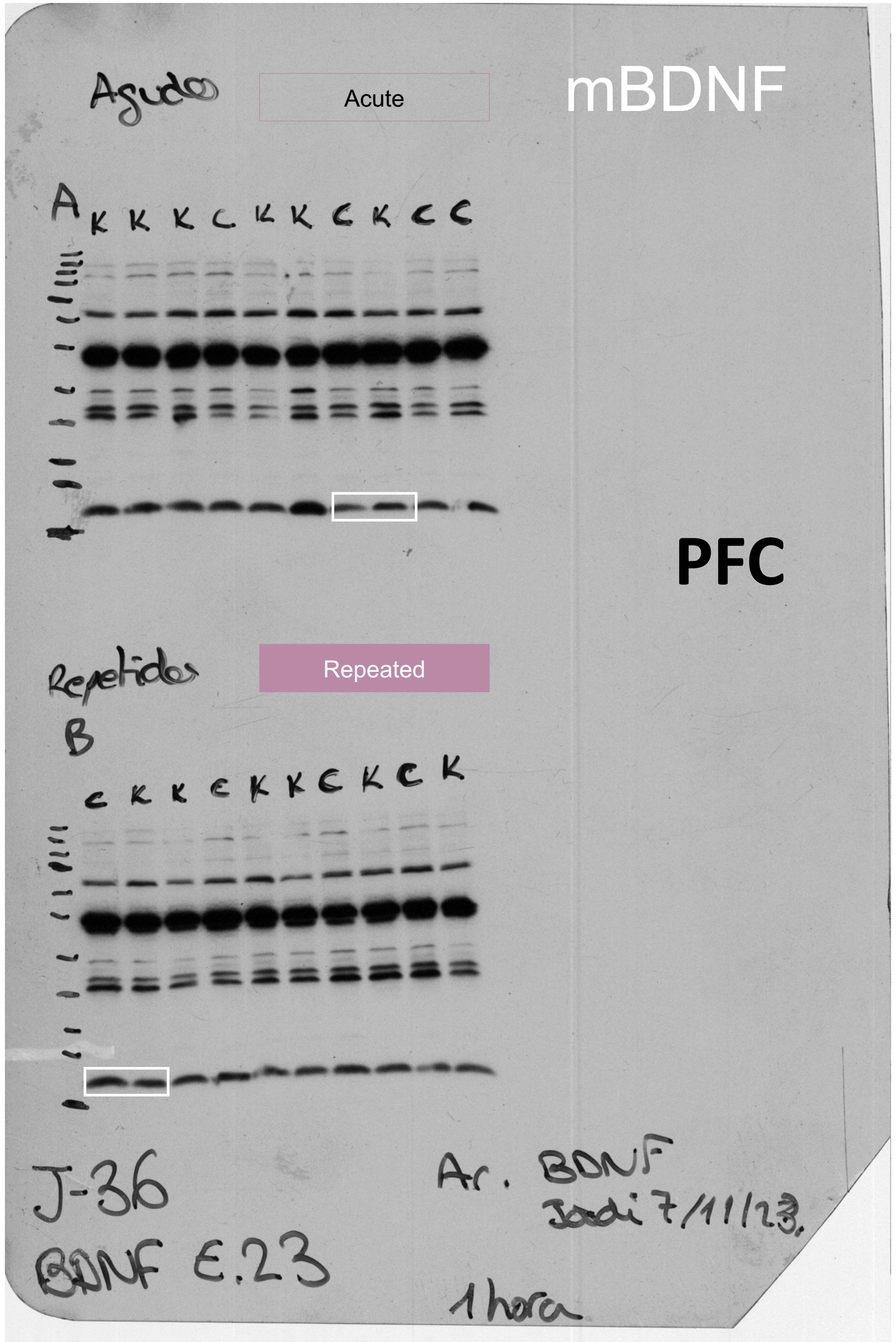

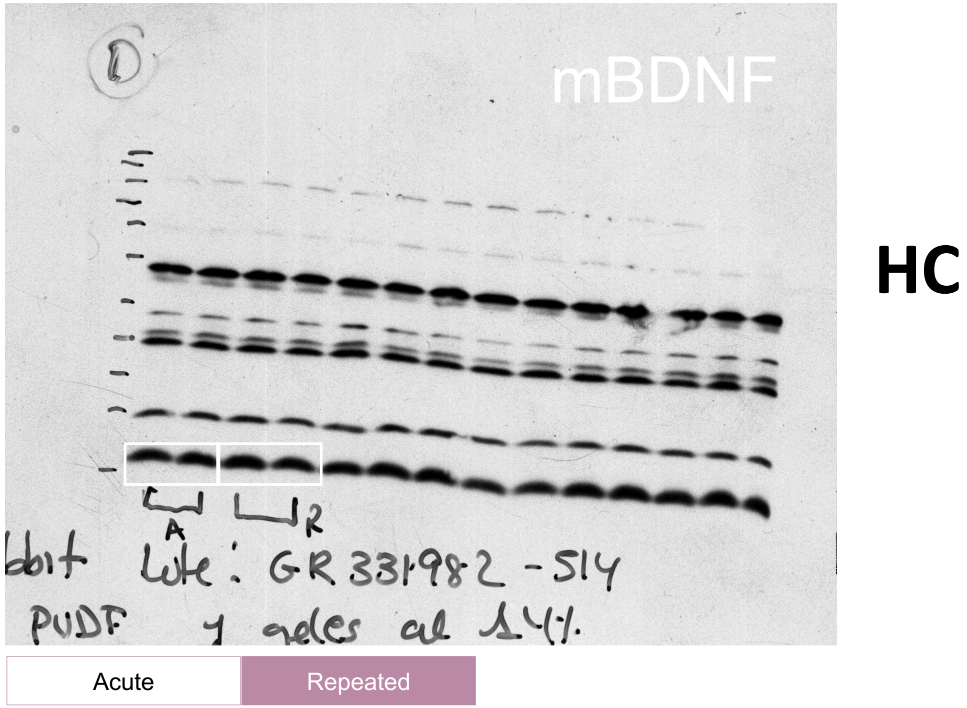

Acute and repeated samples loaded in the same gel

FIGURE S1. Unprocessed western blot images from which representative immunoblots depicting the labeling of mBDNF in prefrontal cortex (PFC) and hippocampus (HC) are shown in Fig. 3c.

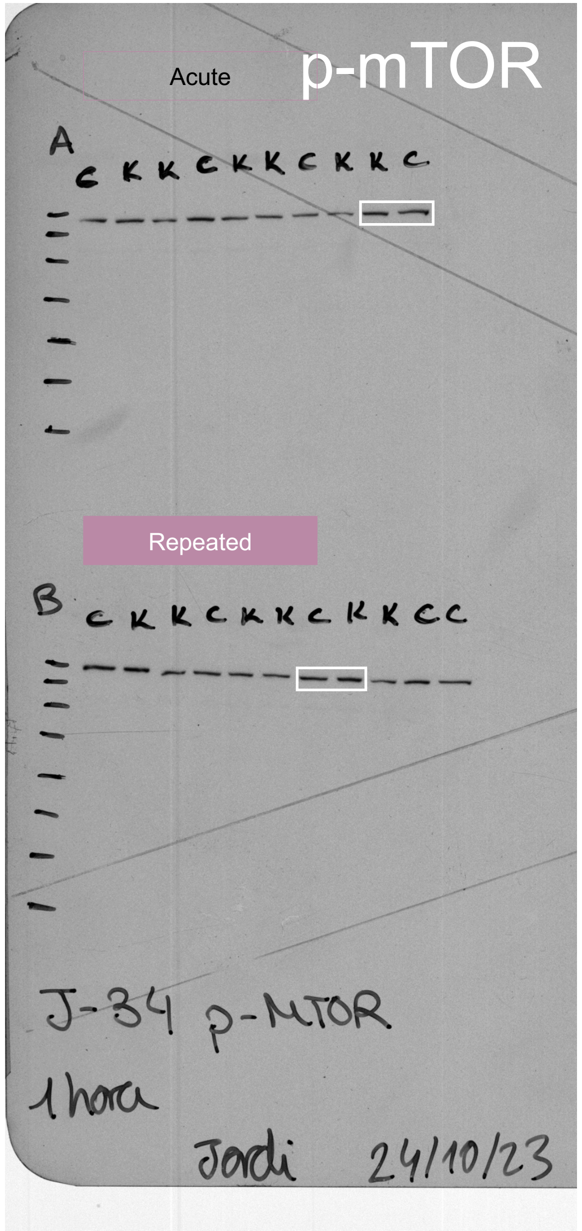

PFC

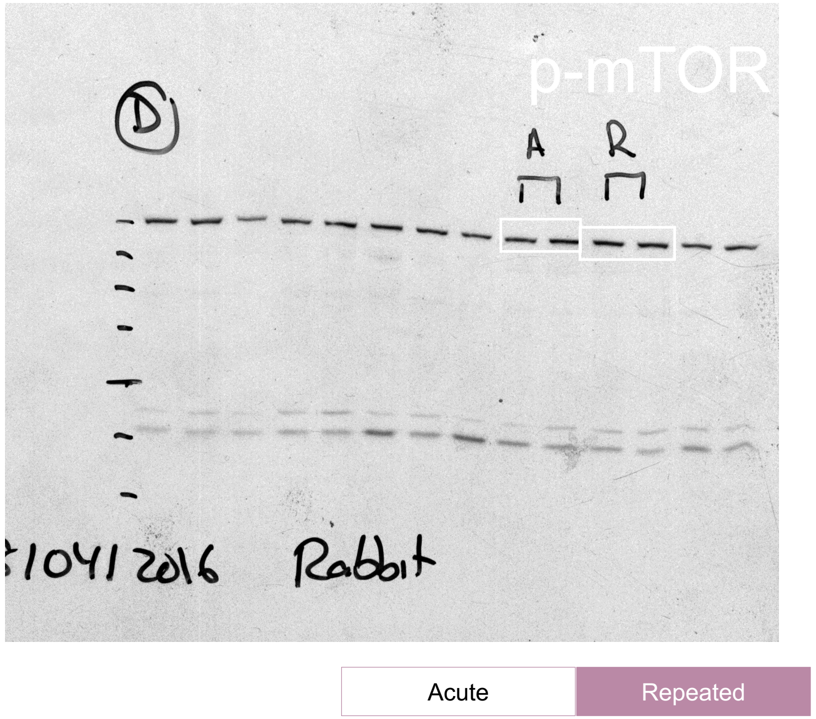

HC

Acute and repeated samples loaded in the same gel

FIGURE S2. Unprocessed western blot images from which representative immunoblots depicting the labeling of p-mTOR in prefrontal cortex (PFC) and hippocampus (HC) are shown in Fig. 3c.

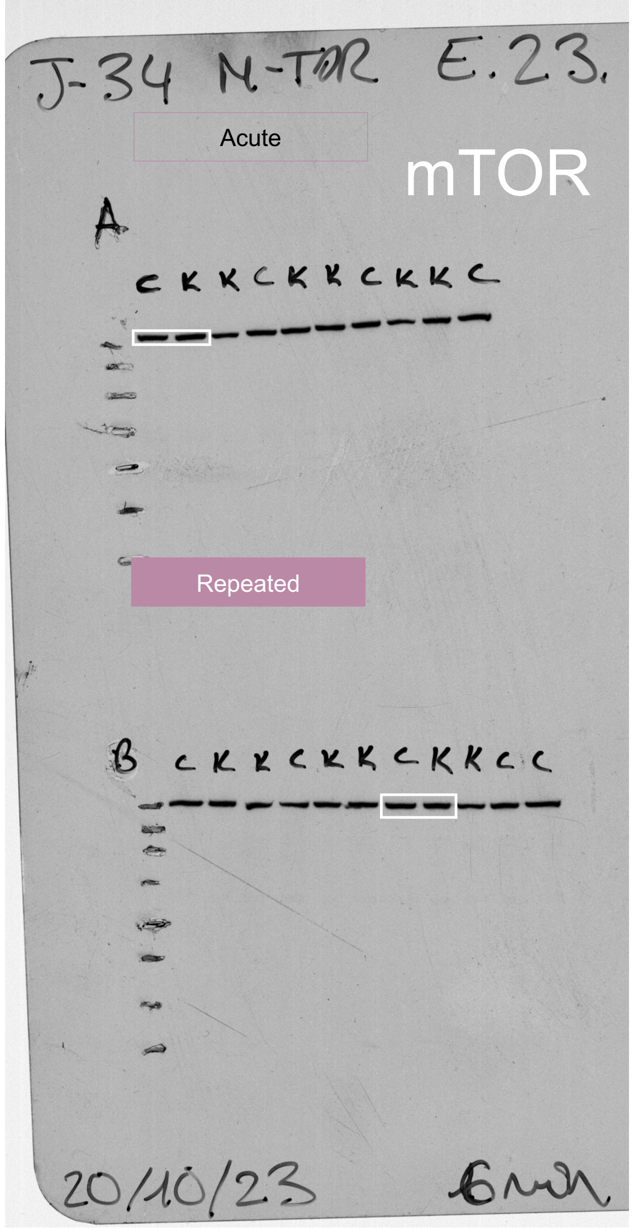

PFC

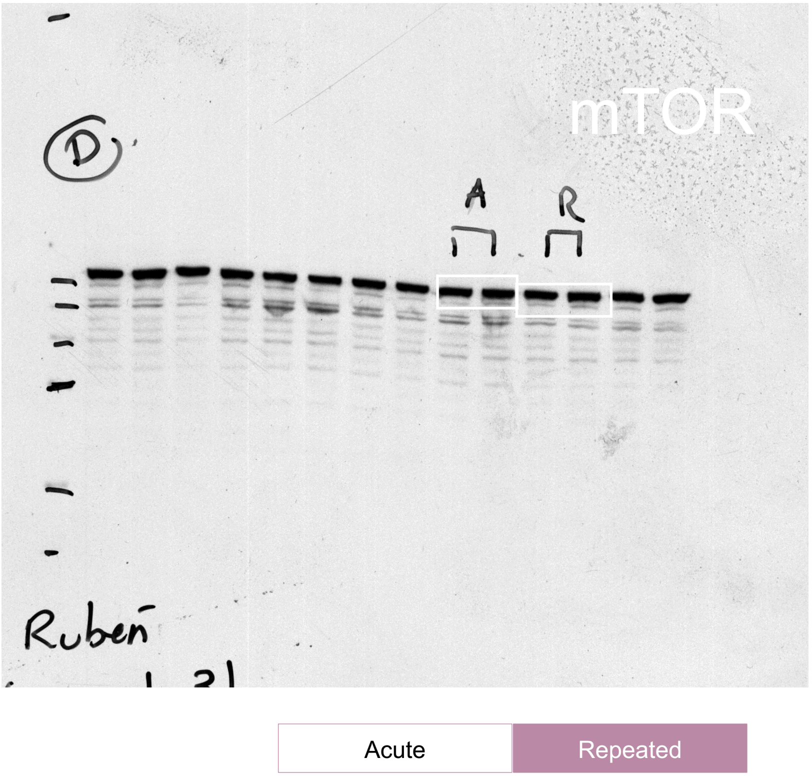

HC

Acute and repeated samples loaded in the same gel

FIGURE S3. Unprocessed western blot images from which representative immunoblots depicting the labeling of mTOR in prefrontal cortex (PFC) and hippocampus (HC) are shown in Fig. 3c.

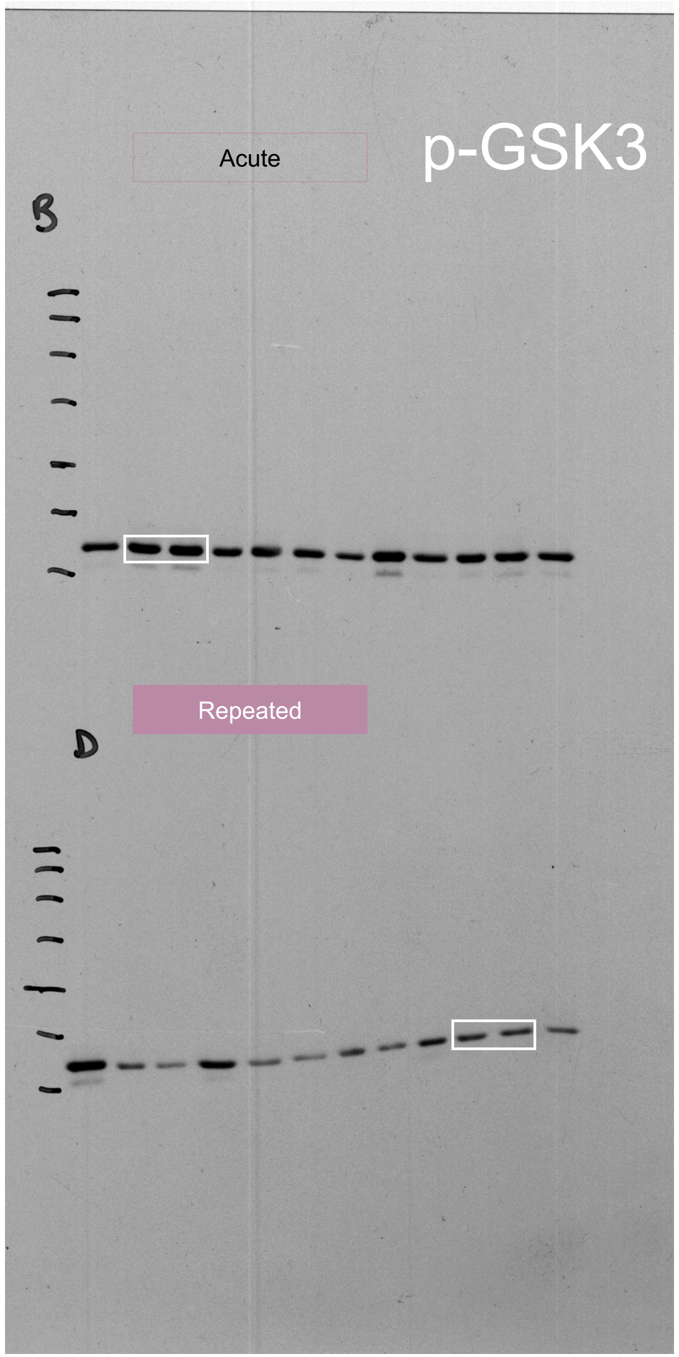

PFC

HC

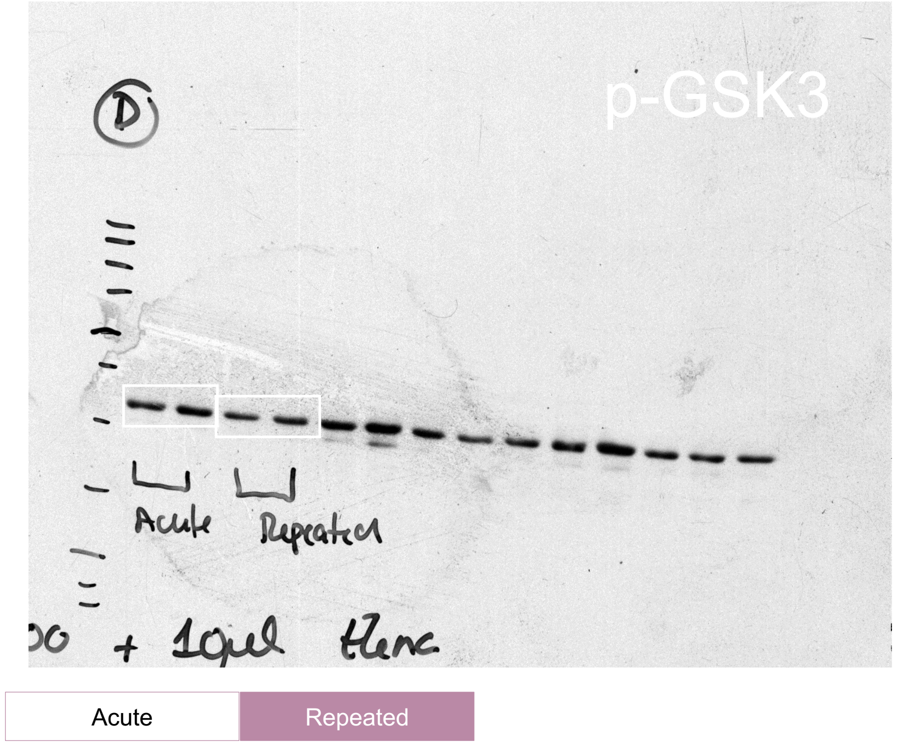

Acute and repeated samples
loaded in the same gel

FIGURE S4. Unprocessed western blot images from which representative immunoblots depicting the labeling of p-GSK3 in prefrontal cortex (PFC) and hippocampus (HC) are shown in Fig. 3c.

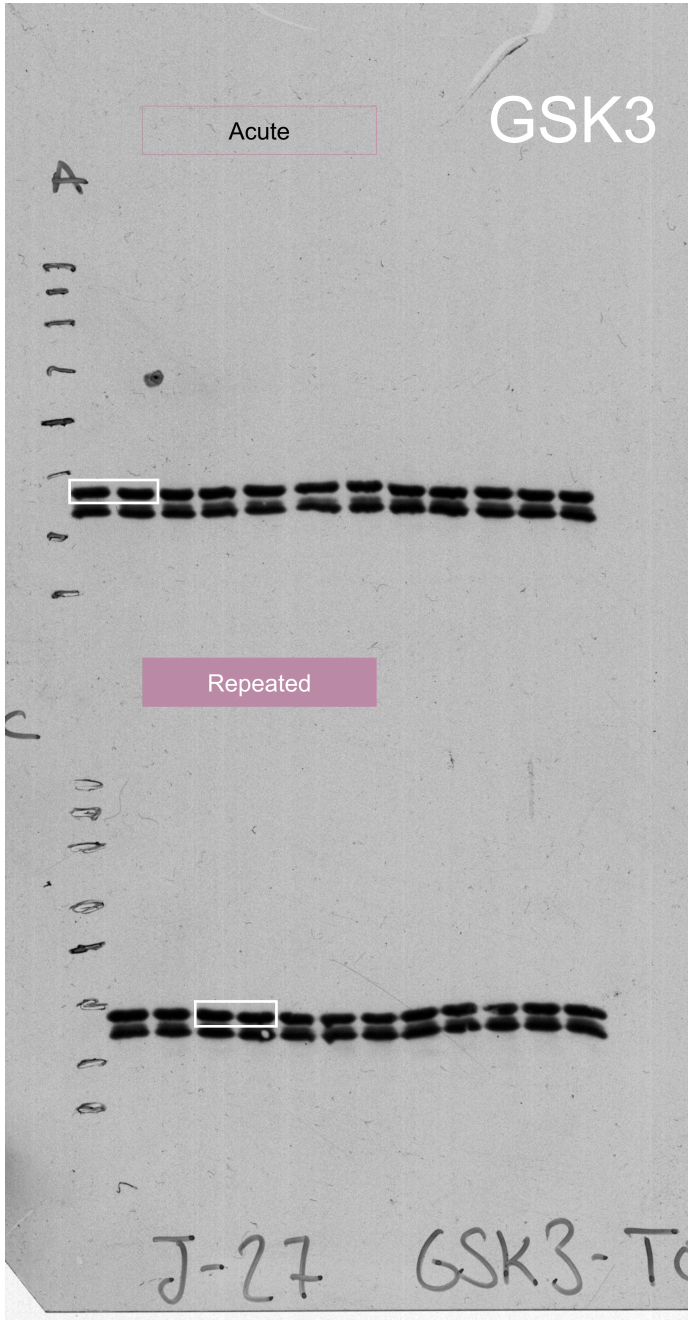

PFC

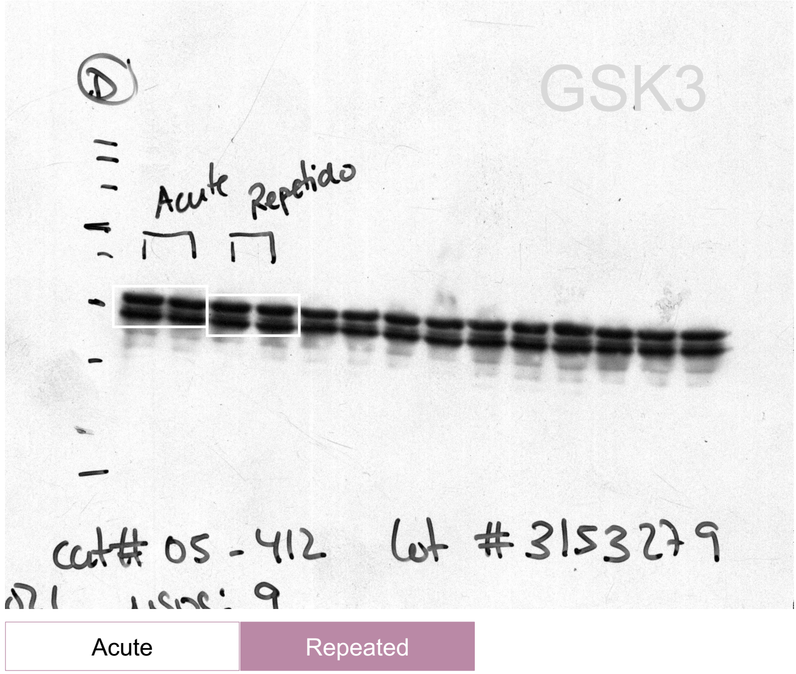

HC

Acute and repeated samples loaded in the same gel

FIGURE S5. Unprocessed western blot images from which representative immunoblots depicting the labeling of GSK3 in prefrontal cortex (PFC) and hippocampus (HC) are shown in Fig. 3c.

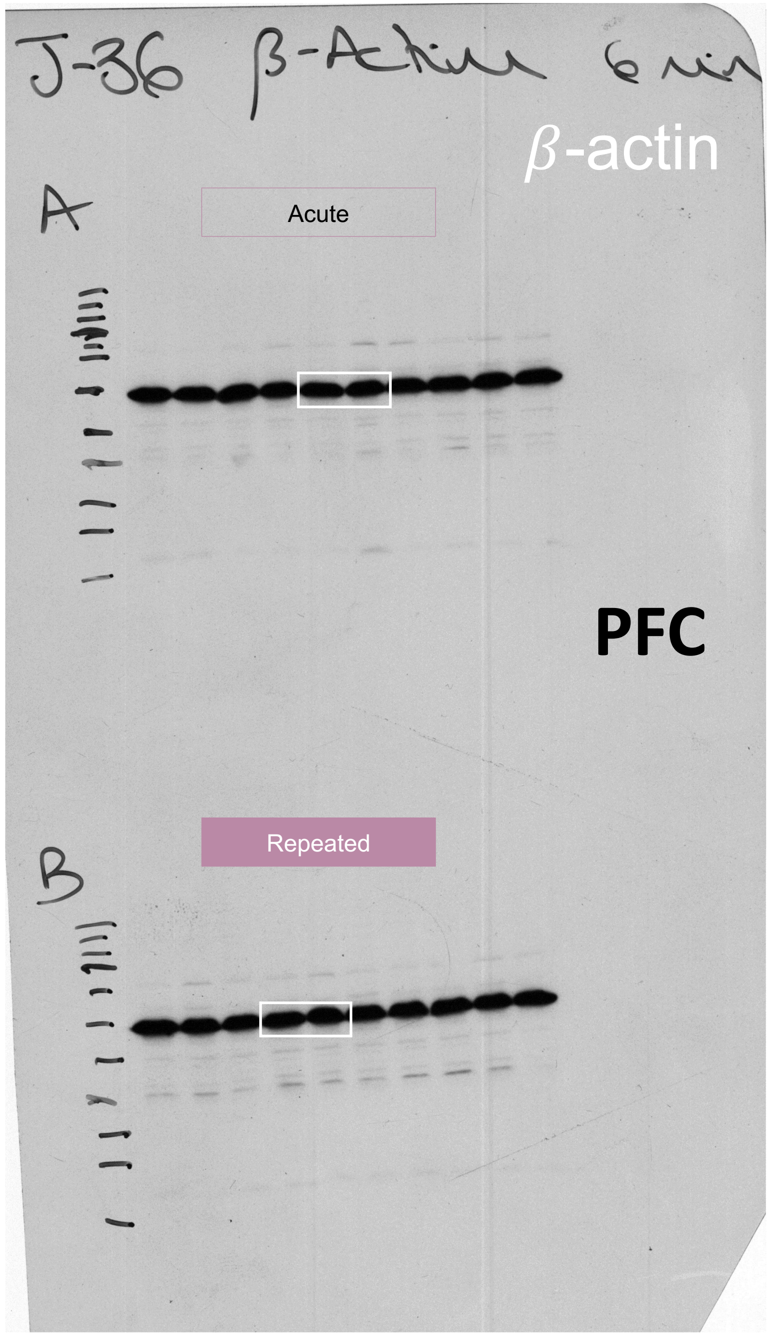

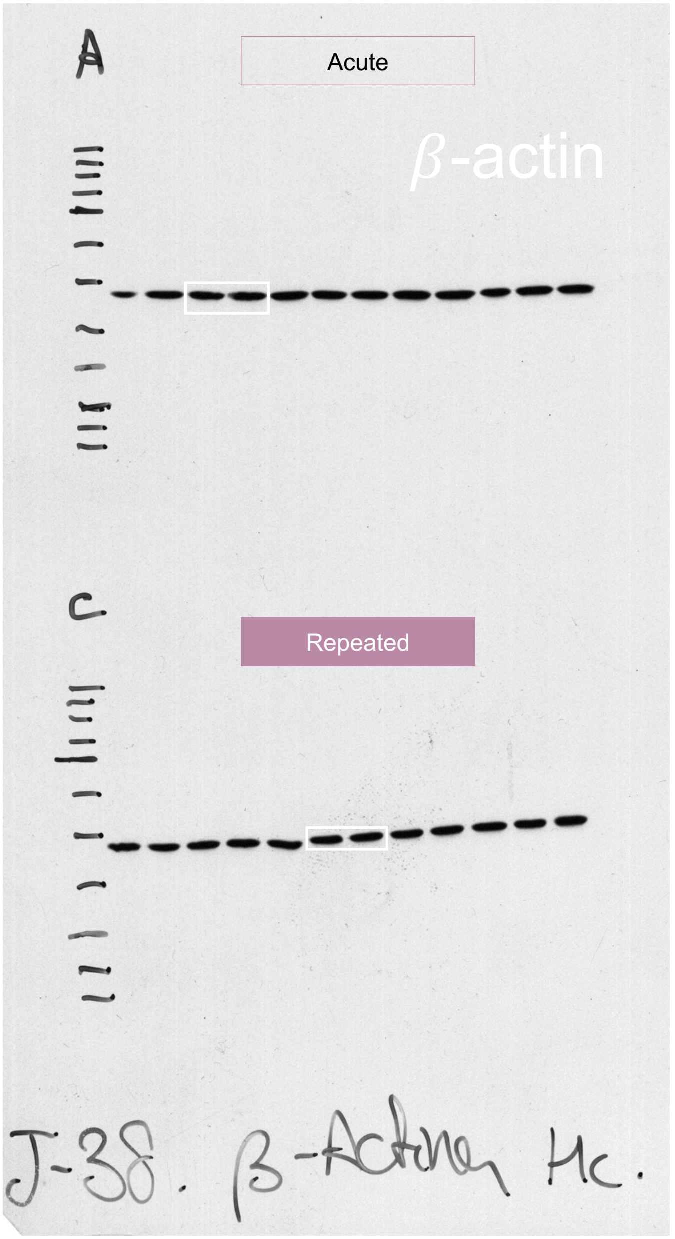

HC

FIGURE S6. Unprocessed western blot images from which representative immunoblots depicting the labeling of β-actin in prefrontal cortex (PFC) and hippocampus (HC) are shown in Fig. 3c.

Acute

C

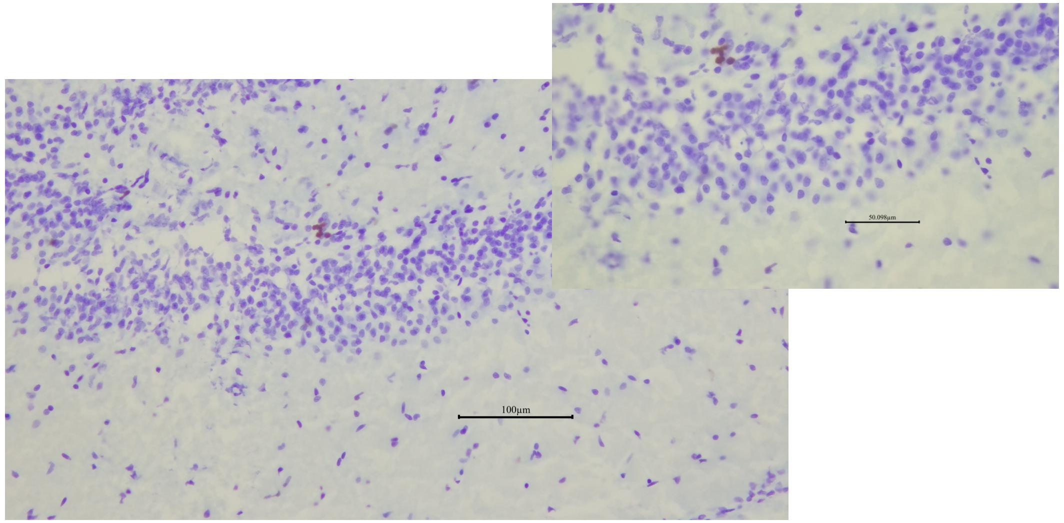

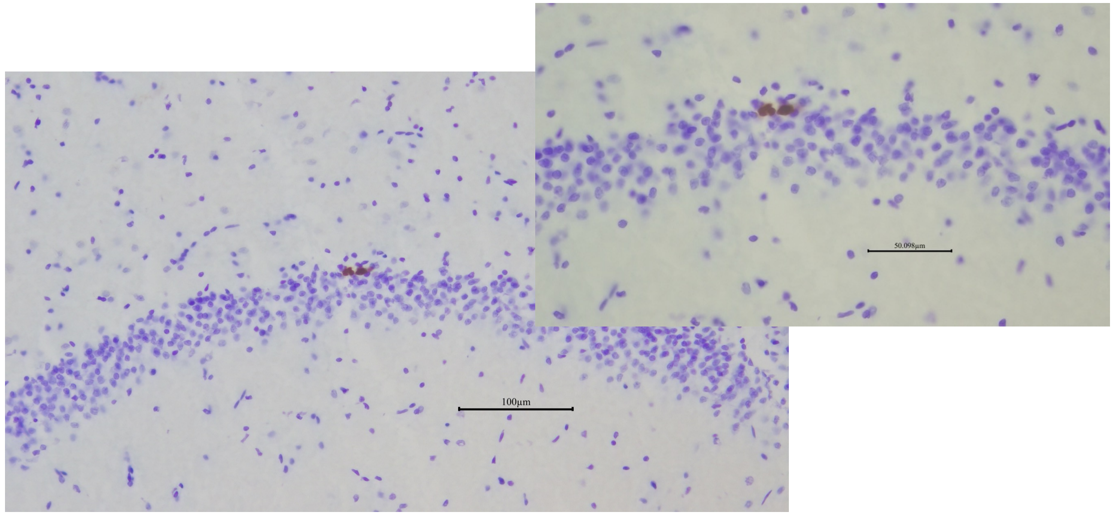

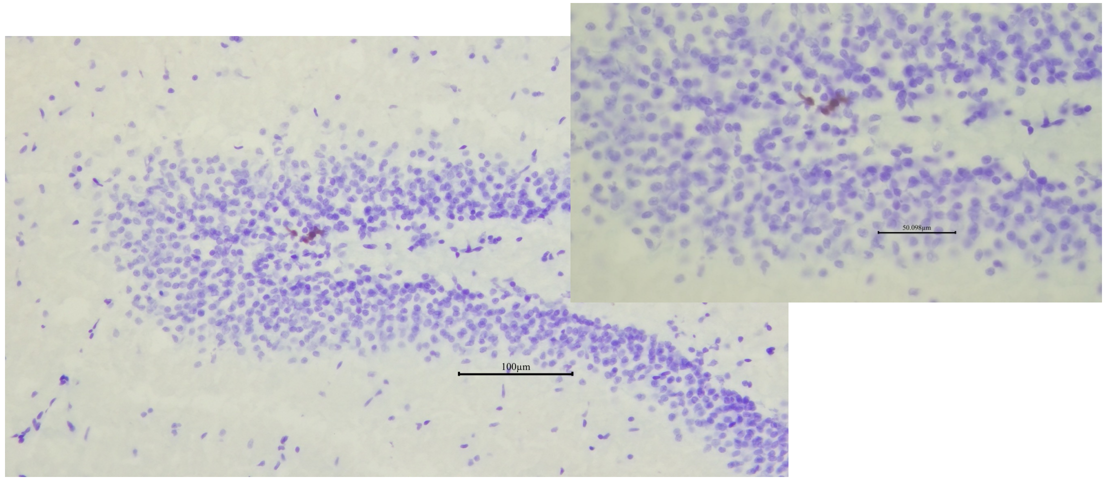

FIGURE S7. Representative images of Ki-67 +cells (brown labeling in the blue granular layer) taken with a light microscope using a 40x objective lens for acute saline-treated rats (control group). A magnified window is shown at 63x. Scale bar: 100 µm. These images are complementary (from 3 different animals) to the ones shown in Fig. 3c.

Acute

Ket

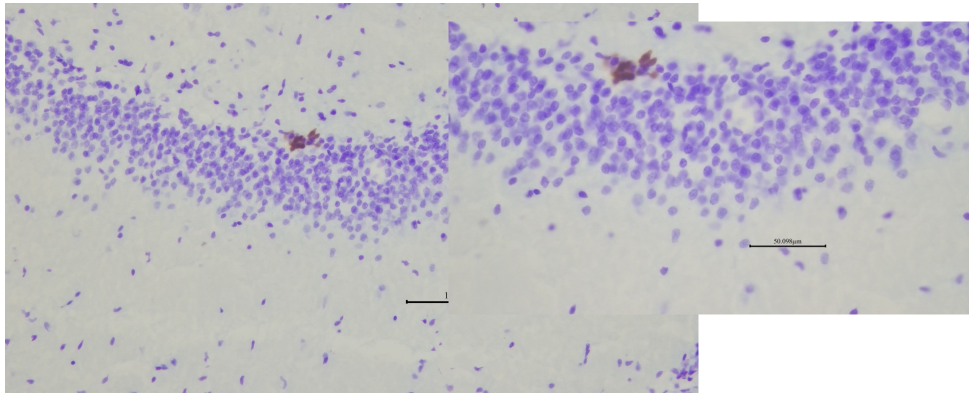

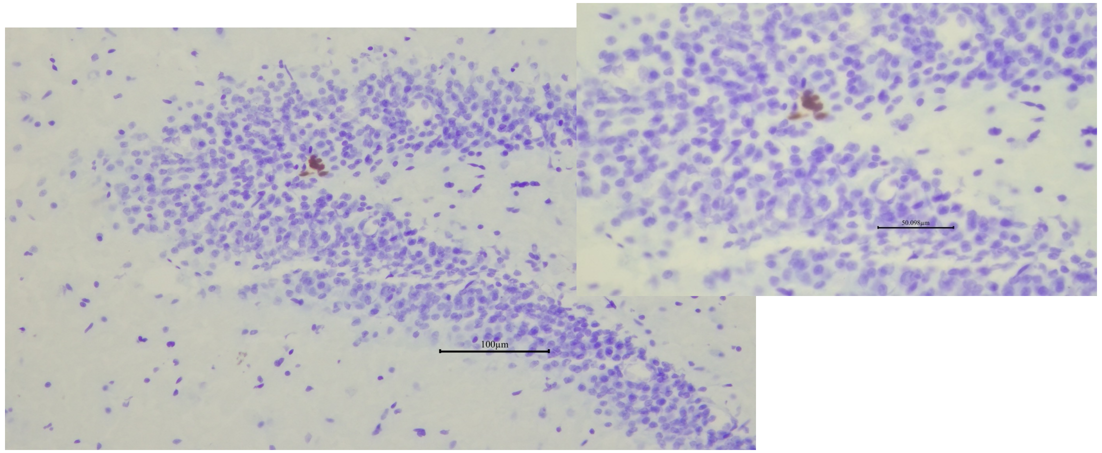

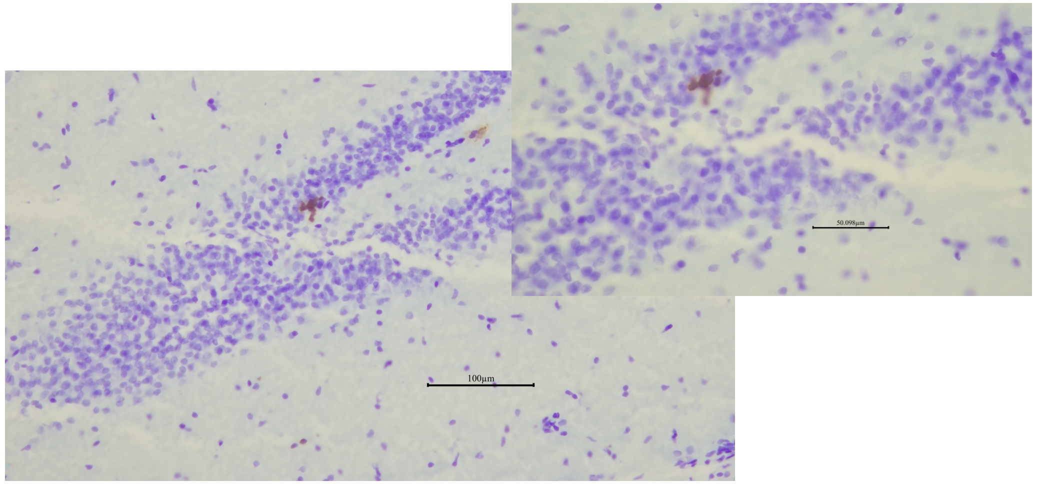

FIGURE S8. Representative images of Ki-67 +cells (brown labeling in the blue granular layer) taken with a light microscope using a 40x objective lens for acute ketamine-treated rats. A magnified window is shown at 63x. Scale bar: 100 µm. These images are complementary (from 3 different animals) to the ones shown in Fig. 3c.

Repeated

C

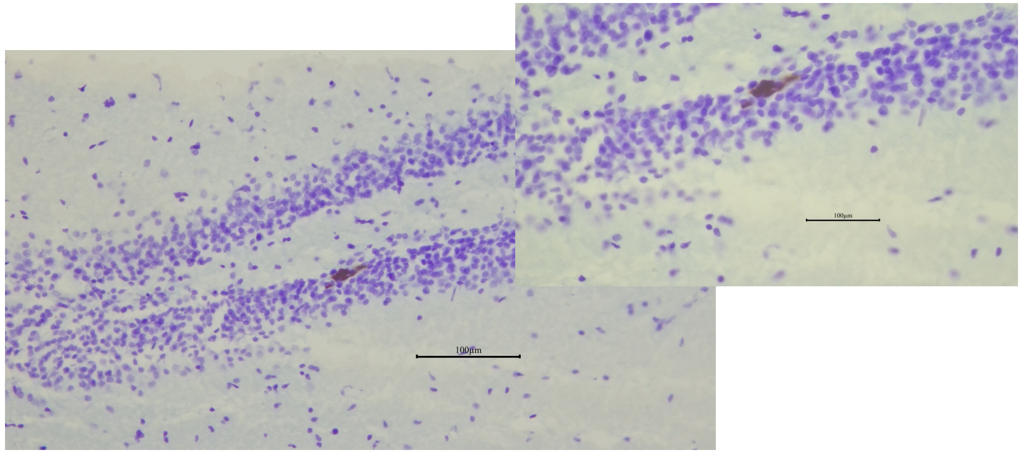

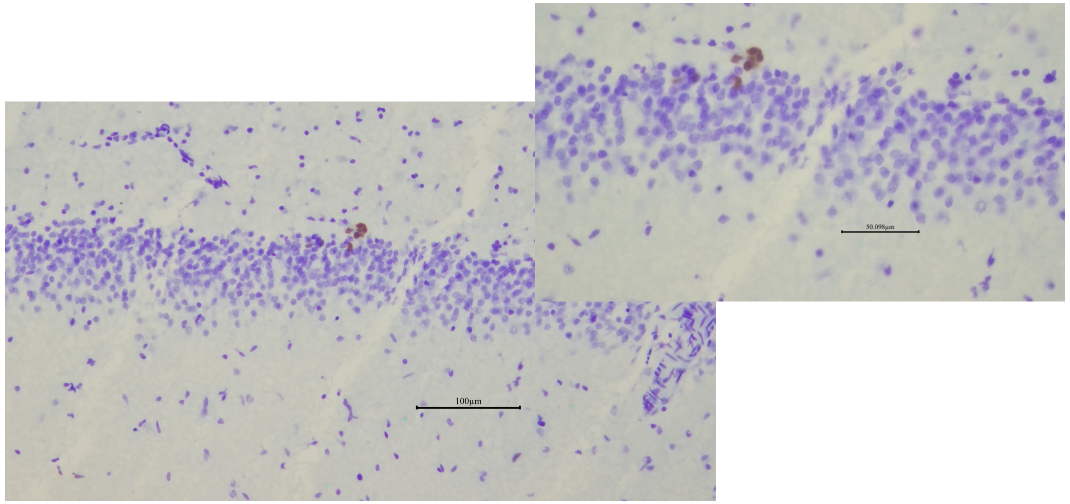

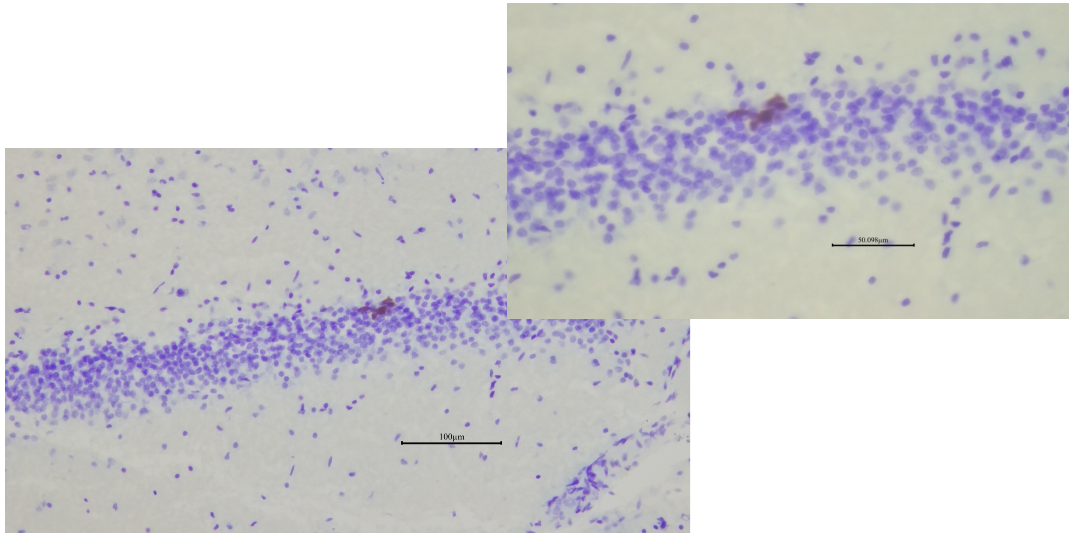

FIGURE S9. Representative images of Ki-67 +cells (brown labeling in the blue granular layer) taken with a light microscope using a 40x objective lens for repeated saline-treated rats (control group). A magnified window is shown at 63x. Scale bar: 100 µm. These images are complementary (from 3 different animals) to the ones shown in Fig. 3c.

Repeated

Ket

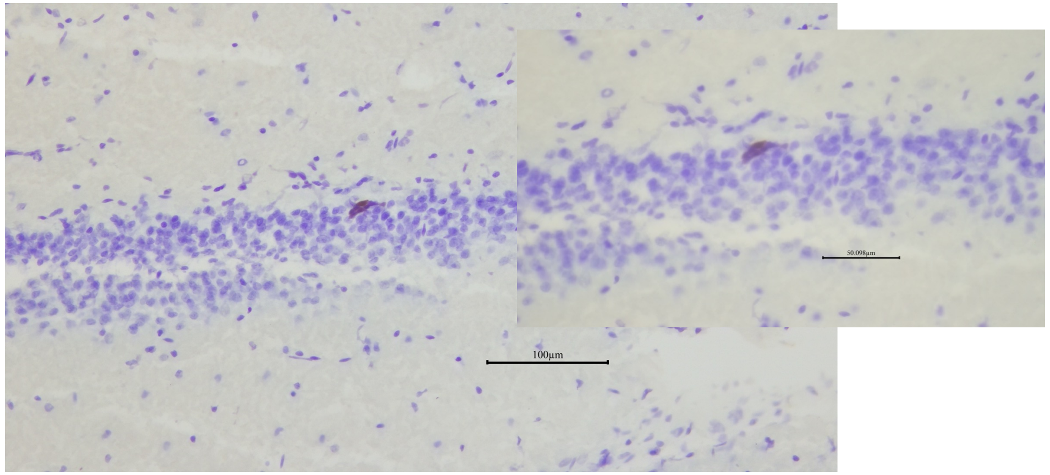

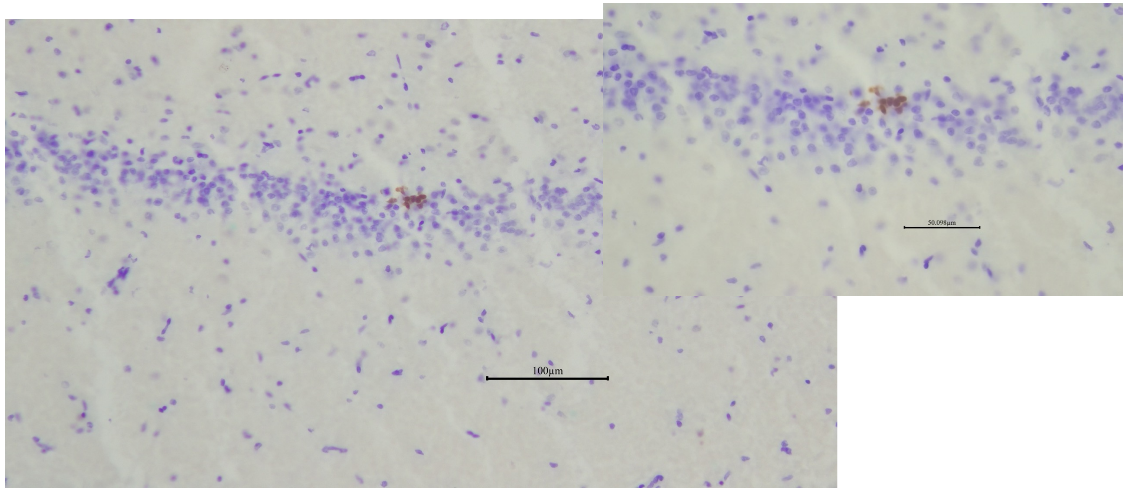

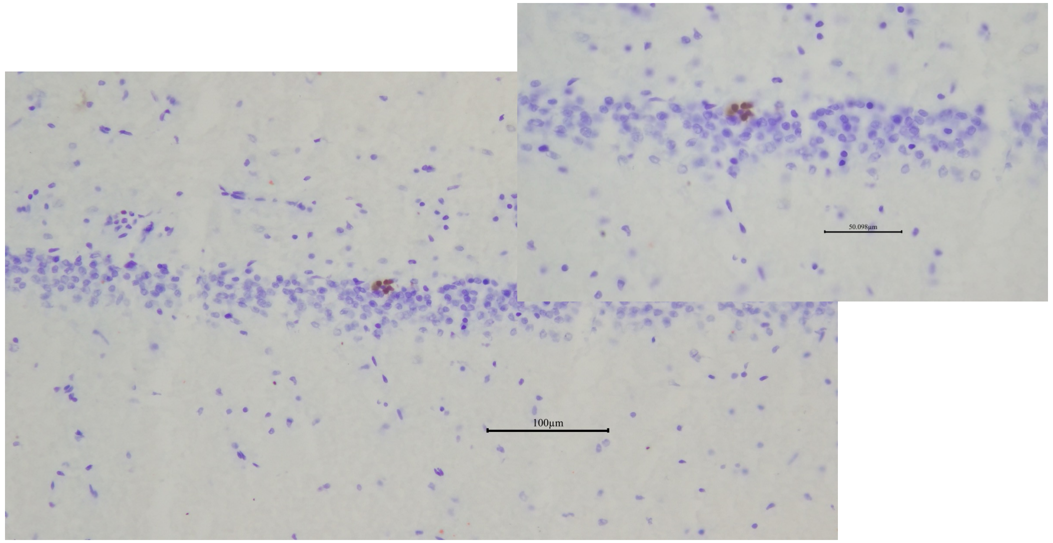

FIGURE S10. Representative images of Ki-67 +cells (brown labeling in the blue granular layer) taken with a light microscope using a 40x objective lens for repeated ketamine-treated rats. A magnified window is shown at 63x. Scale bar: 100 µm. These images are complementary (from 3 different animals) to the ones shown in Fig. 3c.
